# A comparative analysis and review of how national guidelines for chronic disease monitoring are made in low- and middle-income compared to high-income countries

**DOI:** 10.7189/jogh.11.04055

**Published:** 2021-09-04

**Authors:** Elton Mukonda, Maia Lesosky

**Affiliations:** Division of Epidemiology & Biostatistics, School of Public Health & Family Medicine, University of Cape Town, Cape Town, South Africa

## Abstract

**Background:**

Understanding how clinical practice guidelines and recommendations are adopted in high-income and low-income settings will help contextualise the value and validity of recommendations in different settings. We investigate how major guidelines and recommendations are developed for management and monitoring of post-diagnosis treatment for three important chronic diseases: HIV, hypertension and type 2 diabetes mellitus (T2DM).

**Methods:**

Eligible guidelines were searched for using PubMed, Google, and health ministry websites for all three conditions. Only guidelines published from 2010 to 2020 were included. The source of the guidelines, year of most recent guideline, and basis of the guidelines were assessed. Additionally, recommendations, the strength of the recommendation and the quality of the evidence for treatment goals of non-pregnant adults and the frequency of monitoring were also extracted and assessed.

**Results:**

Of the 42 countries searched 90%, 71% and 60% had T2DM, hypertension and HIV guidelines outlining targets for long-term management, respectively. Most T2DM guidelines recommend an HbA1c target of ≤7.0% (68%) or ≤6.5% (24%) as the ideal glycaemic target for most non-pregnant adults, while hypertension guidelines recommend blood pressure (systolic blood pressure/diastolic blood pressure) targets of <140/90 mm Hg (94%) and <130/80 mm Hg (6%). Of the identified HIV guidelines, 67% define virological failure as a viral load >1000 copies/mL, with 26%, mostly HICs, defining virological failure as a viral load >200 copies/mL. Recommendations for the frequency of monitoring for any diagnosed patients were available in 18 (55%) of the hypertension guidelines, 25 (93%) of HIV guidelines, and 27 (73%) of the T2DM guidelines. Only a few of the guidelines provide the strength of the recommendation and the quality of the evidence.

**Conclusions:**

Most guidelines from LMICs are adopted or adapted from existing HIC guidelines or international and regional organisation guidelines with little consideration for resource availability, contextual factors, logistical issues and general feasibility.

Effective management of chronic diseases is multifactorial and includes health system components related to prevention, effective screening and diagnosis, pharmacological/non-pharmacological treatment strategies and then lifelong, routine monitoring for continued patient health. The often long-term nature of chronic disease, especially when health systems prioritise early diagnosis, results in considerable strain on health service resources. Consequently, increasing burdens of chronic diseases pose huge challenges in LMIC health systems, which have been developed mainly for acute conditions and single communicable disease vertical care systems (eg, Malaria, or HIV) and so are ill-equipped to deal with the costs of disease management and long-term treatment required for effective care of people with chronic conditions [1,2]. Depending on age, sex and chronic condition profile, diagnosis with a chronic condition is expected to result in increase in health care services use and costs [3] due to increased physician visits, hospital care, and prescription drugs [4], with these costs lying squarely on the country’s health system, private insurance or individual patients as out-of-pocket payments [5].

Key tools that aid in chronic disease management in both policy and practice, from clinical decisions at the bedside, and patient choices, to governance of health facilities and spending by government and health insurers are clinical practice guidelines [6]. International and national clinical practice guidelines are meant to contain pragmatic and standardized actions or recommendations to be taken by various stakeholders in order to improve the quality of care and subsequently patient health outcomes [7-10]. Guidelines based care has been promoted given that successful implementation often leads to reductions in both mortality and morbidity [7], and costs [11]. In spite of possible benefits for clinical providers to adhere to guideline-based care [7-10], most measures of implementation fidelity and adherence to guidelines are poor [8,10]. In LMICs factors such as poor health literacy, limited health budgets, limited facilities, inadequate clinical expertise and personnel, poor drug supply and out-of-pocket health expenditures are thought to predominate [12], whereas in HIC, complexity of guidelines, poor access to the guidelines for clinicians, time and resource constraints have also been cited as reasons for non-adherence to clinical practice guidelines [10]. In both settings a combination of health system factors and individual factors (both provider and patient) have a role, as does how the guidelines were developed [13-15].

Due to a paucity of well-designed guidelines in LMICs, there is a general reliance on guidelines developed in HICs by third-party guideline production organisations (eg, UK’s National Institute for Health and Clinical Excellence), professional associations (eg, American Diabetes Association) and government health departments or those developed by international organisations (eg, World Health Organisation) [13]. However, the extent to which guidelines developed for HIC settings and patient representation, are relevant to LMIC settings, is a major challenge [9]. Comparisons of guidelines for T2DM and hypertension in LMIC vs HIC settings found that most LMIC guidelines were lacking in terms of applicability, clarity and socioeconomic contextualization as they were adapted from existing HIC guidelines [16,17], casting light on the difficulty of high implementation fidelity in such settings.

This analysis describes how major guidelines, particularly national clinical care recommendations are developed, and summarises the strength of evidence, comparing LMIC and HIC settings for three important chronic diseases: HIV, hypertension and type 2 diabetes mellitus (T2DM). The focus on these chronic conditions is based on how they are a major cause for concern for low- and middle- income countries (LMICs) [18,19]. Specifically, the direct and indirect relationships between cardiovascular disease, hypertension, and T2DM with HIV and antiretroviral therapy (ART) [20], along with rapid urbanisation, and dietary and lifestyle changes [21], are projected to lead to substantial increases in the prevalence of non-communicable diseases rates in LMIC, but particularly in countries with high HIV burden. Currently, sub-Saharan Africa carries almost two-thirds of the HIV burden globally [22] and is predicted, along with Asia, to see the greatest increase in T2DM prevalence by 2045 [23]. Both regions are also among the regions with the highest prevalence of hypertension, a major modifiable risk factor for cardiovascular disease which is the leading cause of death worldwide [24].

The focus of this study is on the guidelines for the post-diagnosis management and monitoring of disease due to the significant health system cost of long-term monitoring. Specifically, the study will focus on the use of biomarkers, which in the broadest definition, are physical measures of individual health commonly used to monitor disease progression, treatment efficacy, and to determine risk of progression or complication [11]. For the three chronic diseases in question, biomarker-based monitoring is conducted using nearly direct and immediate measures of disease like viral load (VL) for HIV and blood pressure (Systolic blood pressure/Diastolic blood pressure) for hypertension, or indirect, medium term markers; Haemoglobin A1c (HbA1c) for T2DM.

## METHODOLOGY

### Search strategy

Searches for guideline documents were conducted on PubMed, Google, and health ministry websites. The terms ‘diabetes’, ‘hypertension’ or ‘HIV/AIDS’, combined with ‘guideline’ or ‘recommendations’ or ‘consensus’, and the country names were used to identify guideline documents. Country selection was based on the classifications by income level, as given by the 2020 World Bank classifications [25]. As a starting point, the countries identified as having guidelines for T2DM or hypertension by Owolabi et al [16,17] were chosen. These included; LIC: Afghanistan, Ethiopia, Liberia, Malawi, Tanzania, Uganda, Zimbabwe; Lower-MIC: Ghana, India, Kenya, Nigeria, the Philippines, Sri Lanka, Swaziland, Zambia; Upper-MIC: Botswana, Brazil, China, Colombia, Fiji, Jamaica, Libya, Malaysia, Mexico, South Africa, Turkey; HIC: Argentina, Australia, Bahrain, Bermuda, Canada, Greece, Hong Kong, Korea Republic, New Zealand, Scotland, Singapore, Sweden, United Kingdom, U.S.A. Additionally, guidelines produced for each condition by regional (Europe and Latin America) or global organisations (eg, World Health Organisation, International Diabetes Federation) were also included, with only recommendations or guidelines published between 2010-2020 included. For guidelines published multiple times during this period, the most recent guideline was summarised with reference to older guidelines. Guidelines that were not in English were translated using Google translate.

### Data extraction

As guidelines vary in their scope, we focused on sections that covered the treatment targets and the frequency of biomarker monitoring among non-pregnant adults for each of the three conditions. The specific recommendations, the strength of the recommendations and the quality of the evidence were all extracted and retained for further analysis.

### Analysis

The identified guidelines were assessed for the basis of the guidelines (were they specifically based on international guidelines or consensus documents), specific recommendations for treatment targets of non-pregnant adults and the frequency of biomarker monitoring, the strength of the recommendations and the quality of the evidence. Recommendations focusing on special populations were excluded from this analysis. All results were presented with frequencies and percentages. Univariate analysis was carried out to assess differences in selected variables by income classification, with a *P* value <0.05 deemed significant. All analyses were done in R version 3.6 (R Core Team, Vienna, Austria).

## RESULTS

A total of 89 full text guidelines from the 40 countries, and 8 full-text guidelines from international/regional organisations were reviewed. Specifically, 37 T2DM guidelines, 33 hypertension guidelines and 27 HIV guidelines were reviewed for this study. Less than half of the countries had national guidelines for all three conditions (n = 18, 42.9%), while 10 (23.8%) had guidelines for 2 of the conditions and a third only had guidelines for only one condition (n = 14, 33.3%). Fully, 85% (n = 34) of the 40 countries included had guidelines for T2DM and included specific recommendations for long-term management. Nearly three-quarters of countries (n = 30, 71.4%) had hypertension guidelines that specified treatment targets, and just over half of the countries (n = 25, 60%) had HIV guidelines that also outlined biomarker-based treatment targets. A summary of the country of origin, year of publication, source of the guidelines, recommendations, the strength of the recommendations, and the quality of the evidence for control targets, and the frequency of monitoring are reported in Tables S1-S3 in the **Online Supplementary Document****.**

### Treatment targets

**Figure 1:**
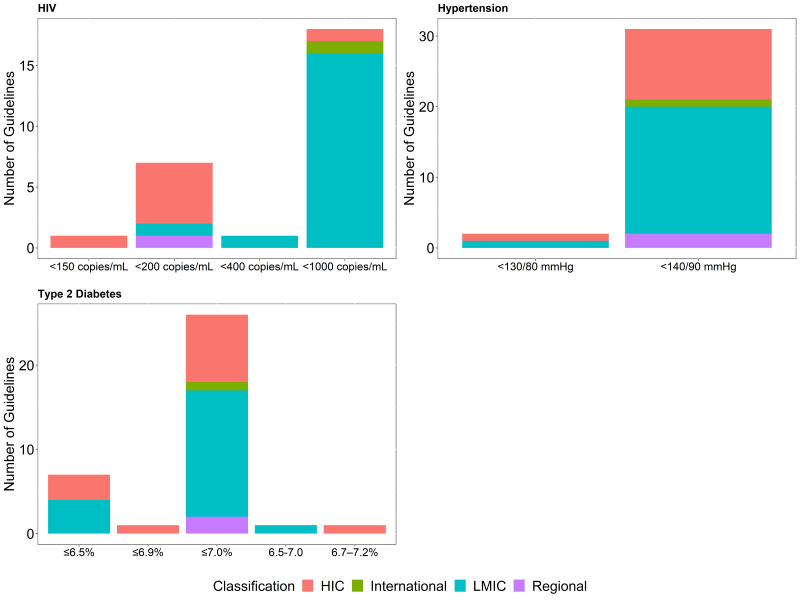
Treatment targets for HIV, hypertension and type 2 diabetes by the income group of the country adopting the recommendation/guideline.

Treatment targets for all three conditions vary across guidelines as shown in **Figure 1**. Most T2DM guidelines recommend an HbA1c target of ≤7.0% (n = 26, 72.2%) or ≤6.5% (n = 7, 19.4%) as the ideal glycaemic target for non-pregnant adults. Hypertension guidelines recommend blood pressure (systolic blood pressure/diastolic blood pressure) targets of <140/90 mm Hg (94% of the guidelines) and <130/80 mm Hg (6% of the guidelines) for the same population showing remarkable consistency across countries and settings. For HIV, approximately two-thirds (n = 18, 66.7%) of the identified guidelines define virologic failure as HIV viral load >1000 copies/mL, with 26% (n = 7/27), mostly HICs, defining virologic failure as a HIV viral load >200 copies/mL. While there is no discernible relationship between the choice of treatment target and country income classification (HIC vs LMIC) for T2DM (*P* = 0.436), and hypertension (*P* = 0.685), there does appear to be a difference between the HIV virologic failure definition and guideline setting (*P* = 0.001).

Overall, only 14 (42.4%) of the identified hypertension guidelines, including the global and regional guidelines, provide the strength of the recommendation or the quality of the evidence supporting the adoption of the treatment goal, with many of the guidelines being from HICs (9 of the guidelines) or Upper-MICs (3 of the guidelines). For T2DM guidelines, 20 (54%) of the identified guidelines provide the strength of the recommendation or the quality of the evidence supporting the adoption of the glycaemic target, with HICs and Upper-MICs accounting for 18 (90%) of these, while only 10 (37%) of the HIV guidelines provide the quality of evidence supporting the definition of treatment failure. Comparing the availability of the strength of the recommendation or the quality of the evidence in guidelines between HIC and LMIC, 9 (82%) of the hypertension guidelines from HIC provided both as opposed to 3 (16%) LMIC guidelines (*P* < 0.001). All HIV guidelines from HIC provided information on the strength of the recommendation or evidence rating as opposed to 1 guideline from LMIC (*P* < 0.001), while 11 (79%) of the T2DM guidelines from HIC as opposed to 8 (40%) from LMIC (*P* = 0.026).

### Monitoring frequency

In most of the identified guidelines, for all three conditions, recommendations for the frequency of monitoring the relevant biomarkers were different for newly diagnosed patients (on treatment) or patients not meeting targets, and patients meeting targets on stable, unchanging therapy. Recommendations for the frequency of monitoring for any diagnosed patients were available in 18 (55%) of the hypertension guidelines, 25 (93%) of HIV guidelines, and 27 (73%) of the T2DM guidelines. Of these, only 5 (28%) of the hypertension guidelines, 4 (16%) of the HIV guidelines and 11 (41%) of the T2DM guidelines provide the strength of the recommendation and the quality of the evidence.

**Figure 2:**
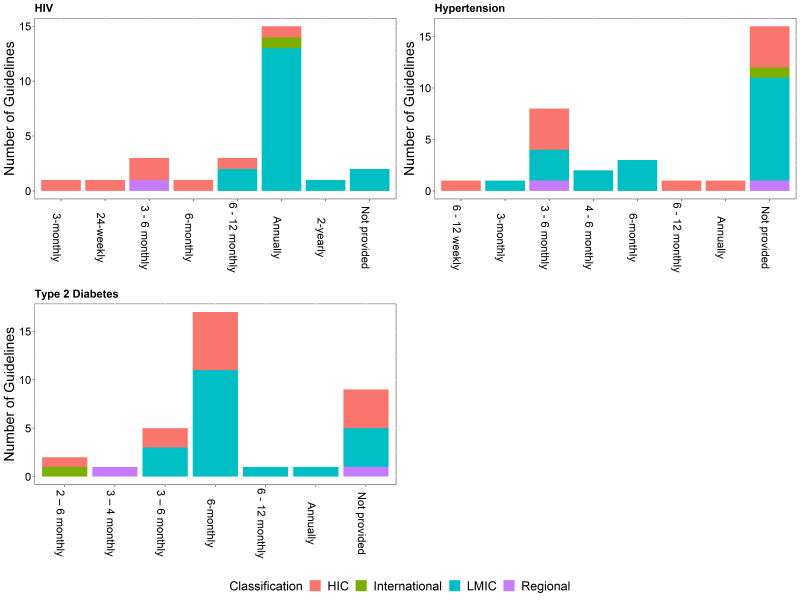
Frequency of monitoring for stable patients with HIV, hypertension and type 2 diabetes by the income group of the country adopting the recommendation/guideline.

**Figure 3:**
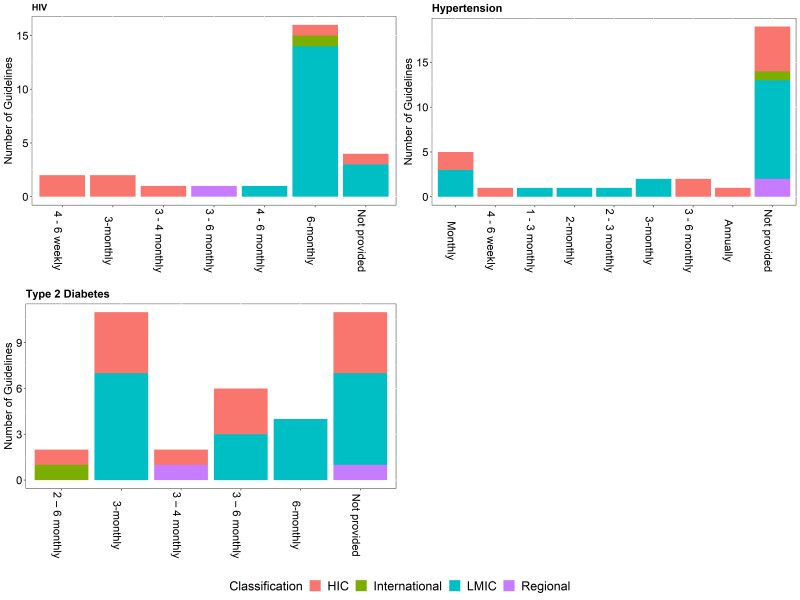
Frequency of monitoring for newly diagnosed patients or patients not meeting targets with HIV, hypertension and type 2 diabetes by the income group of the country adopting the recommendation/guideline.

Newly diagnosed patients or patients not meeting targets often require more frequent monitoring than for patients meeting targets as there is a need to assess a patient’s response to treatment and possible adverse effects before achieving control [11]. This difference is reflected in most of the identified guidelines, for all three conditions. As an example, recommendations for monitoring HbA1c among newly diagnosed patients or patients not meeting targets ranges from 2 to 6 months, while stable patients can be monitored between 2 months and 1 year (**Figure 2** and **Figure 3**). Comparing the recommended frequency of monitoring for HIC vs LMIC, there is no significant difference for T2DM and hypertension, while there appears to be a difference between income classification and the recommended frequency of monitoring of HIV viral load for both newly diagnosed patients (*P* = 0.005) and stable patients (*P* = 0.017).

### Strength of the recommendations and the quality of evidence

When provided, the rating scales of both strength of the recommendation and the quality of evidence often differed depending on guideline source(country) and condition. The strength of the recommendation is dependent, primarily, on the quality of the clinical and economic evidence supporting the recommendation [26]. Other considerations include patient values and preferences, applicability of published evidence to the target population, the balance of benefits and harms of the options, and economic costs [13]. Generally, the grading of recommendations ranges from strong, conditional, optional, or weak. Consistent, patient-oriented evidence from systematic reviews, meta analyses on high-quality RCTs with a low risk of bias, or high-quality individual RCTs are generally associated with the strong recommendations, while consensus guidelines, usual practice, expert opinion and disease-oriented evidence are generally associated with weak recommendations. The glycaemic targets, HbA1c <7.0% and HbA1c <6.5% both had strong recommendations in T2DM guidelines, based on high quality evidence, as did blood pressure targets <130/80 mm Hg and <140/90 mm Hg in hypertension guidelines. The most common definitions of viral failure, VL>200 copies/mL and VL>1000 copies/mL, both had a strong recommendation though the latter is based on low-quality evidence. For all three conditions, the frequency of monitoring in most of the guidelines was based on consensus and expert opinion and had weak recommendations.

## DISCUSSION

By focusing on treatment targets and the frequency of monitoring after initiating treatment, we have provided valuable insight into the differences and similarities of the recommendations adopted in clinical practice guidelines in HICs and LMICs for the management of HIV, hypertension and T2DM. Moreover, this study has also been able, in part, to identify how current recommendations were arrived at for the three conditions. Treatment targets are largely based on systematic reviews and meta analyses on high-quality RCTs with a low risk of bias, or high-quality individual RCTs, often on populations from high income settings. The frequency of monitoring is often based on expert opinion or clinical consensus with little to no consideration made for factors like genomics, socioeconomic context or resource availability despite significant disparities among developed and developing countries. For all three conditions, there are questions concerning the choice of target and thresholds for treatment failure, while validation of the frequency of monitoring in different settings has been the subject of ongoing research.

### Treatment targets

There is general agreement on the target for glycaemic control with most guidelines recommending HbA1c <7.0%, while a few recommend a more stringent target of HbA1c <6.5%. In all the recommendations for treatment targets, allowance was made factors like age, risk of hypoglycaemia, duration of diabetes and comorbidities. Targets for glycaemic control are generally based on the same randomised trials, the United Kingdom Prospective Diabetes Study (UKPDS,1977-1997), the Action to Control Cardiovascular Risk in Diabetes (ACCORD, 2001-2005) study, the Kumamoto study (1995), and the Action in Diabetes and Vascular Disease: Preterax and Diamicron MR Controlled Evaluation (ADVANCE, 2008) study [27-30]. In these studies, glycated haemoglobin (A1C) levels below 7.0% were found to be associated with reductions in clinical microvascular and cardiovascular events. The demographics of the study participants, however, suggest that generalization to LMICs may be inappropriate [31]. The UKPDS had 83% of participants with European ethnicity with a median age of 54 years (IQR 48–60 years), while the ACCORD trial had a mean age of 62 with 64.8% being white. Given the variability in HbA1c for factors like age, race, ethnicity and the existence of comorbidities, assigning a fixed target might not be appropriate thereby giving credence to the Diabetes Canada’s recommendation for individualised targets [32].

Targets for monitoring BP were also consistent at <140/90 mm Hg for most countries and regional bodies and were based on evidence from the ACCORD study [30]. The only exception, however, was the most recent recommendation from the American College of Cardiology (ACC) and American Heart Association (AHA) which was more stringent in defining hypertension and the treatment target [33]. The 2017 ACC/AHA guidelines, which recommend systolic BP<130 or diastolic BP<80 mm Hg, was based on evidence from the SPRINT (Systolic Blood Pressure Intervention Trial) which found that adopting the new definition leads to a reduced rate of major adverse cardiovascular events [34]. This, however, has been questioned by many experts. Poulter et al [35]. on behalf of the International Society of Hypertension, questioned the relevance of these guidelines from a global perspective, especially in low- and middle-income countries. Issues raised included the low global control rates (15%) which would be even lower under the ACC/AHA guidelines and an increase in the prevalence of hypertension. Additional concerns raised were the dangers of inappropriately labelling people as hypertensive which may cause anxiety and over-inflated hypertension treatment in low-risk younger people (especially women) and inadvertent drug associated adverse events.

Looking at the impact of adopting the new blood pressure targets on the South African population, Rayner et al [36] estimated that the prevalence of hypertension would increase from 35.1% in South Africa in 2018 under the current definition, to 50.2% if the new definition is applied. This was calculated assuming that the increase in the number of patients with diagnosed hypertension under this new guideline would be the same as the increase by reported in the USA (43%) [37]. Additionally, Rayner et al highlighted how the new targets would lead to increased demands on already overburdened and under-resourced health services. Specifically, it would lead to increases in; health visits to monitor patients, use of antihypertensives to achieve the lower target, use of laboratory services to monitor for adverse effects. Moreover, it would require a major retraining of health workers. In terms of the financial implications, Wang et al [38] mention how this definition also leads to substantial increases in the direct medical costs with China needing an additional US$ 3.6 billion direct medical costs in each fiscal year.

For viral load, most LMICs adopted the WHO recommendation of VL>1000 copies/mL on 2 consecutive viral load measurements as indicative of failure [39]. The chosen target is based on the fact that the risk of HIV transmission and disease progression is very low when viral load is lower than 1000 copies/mL, and that below this threshold, viral blips or intermittent low-level viraemia (50–1000 copies/mL) can occur during effective treatment but have not been associated with an increased risk of treatment failure [40-42]. Intuitively, if LMICs used the definition used by HICs this would also lead to an increase in the number of individuals with treatment failure, hence requiring more frequent monitoring and testing and potentially unnecessary changes in treatment.

### Monitoring frequency

As monitoring frequency recommendations for all three conditions were based on clinical experience or expert consensus, there is a gap in the evidence on the benefits of repeated testing on patient outcomes [43]. There are, however, several studies investigating the evidence on the optimal monitoring strategies and testing intervals for the three conditions in question. For T2D, studies investigating the relationship between frequency of HbA1c monitoring and glycaemic control have found that lower frequency of monitoring generally led to poor glycaemic control [44,45]. For newly diagnosed patients and patients not meeting targets, the optimum testing frequency was between 2-4 months, with a testing frequency greater than 6 months being associated with a deterioration in control [44-47]. Much like for T2DM patients, patients with newly diagnosed hypertension and those not meeting hypertension treatment targets are expected to have more frequent health care facility visits until they are well-controlled. For blood pressure monitoring, there is evidence to suggest that there are no significant true blood pressure changes within a 12-month period for well controlled patients [48-50], however, reviews every 6 months allow for reinforcement of lifestyle changes, assessment of new risk factors, a review of medication adherence and repeat prescriptions [51].

For HIV, frequent VL monitoring has been found to enable early and accurate diagnosis of treatment failure before the development of drug resistance mutations, thereby improving the quality of care that HIV patients receive [52]. Some studies have shown that visit intervals longer than 3 months did not worsen viral load, and that gaps in care of less than 9 months led to non-significant viral load increases [53-55], especially in clinically stable patients with suppressed viral load. It should be noted that most of the studies mentioned here were in high income settings. As such, there is a need for further investigations into different monitoring frequencies in lower- and middle-income settings.

While there is not much difference between the frequency of monitoring HbA1c in T2DM patients and blood pressure in patients with diagnosed hypertension for LMICs vs HIC, intervals chosen for newly diagnosed HIV patients on ART, patients not meeting targets, and patients meeting targets are also wider for LMICs compared to HICs for individuals on ART. The WHO recommendation of monitoring 6 and 12 months after ART initiation then annually once viral suppression is attained has been adopted by many LMICs. This recommendation, however, has been questioned, with Rafie et al [56] arguing that the use of annual intervals rather than 6-monthly intervals likely increased the time patients remained on a failing (standard) regimen by an additional 6 months, thus increasing the risk of developing antiretroviral resistance mutations.

Most HIC guidelines and Upper-MIC, which were developed by professional associations or third-party guideline production organisations, were clear about the source of the recommendations. Recommendations and the quality of evidence were often graded using the Grading of Recommendations, Assessment, Development and Evaluations (GRADE) [57] or the Strength of Recommendation Taxonomy (SORT) [58] tools. The use of the Appraisal of Guidelines Research & Evaluation (AGREE II) instrument [59] to evaluate and appraise the guidelines was also reported. Another tool which was reported is the ADAPTE methodology, which is used when adapting guidelines produced elsewhere for local use and essentially involves taking the best or most appropriate recommendations and repackaging them into a new local guideline [60]. In contrast, it can be difficult to ascertain how recommendations were arrived at in some LICs and Lower-MICs as guidelines were vague about the source of recommendations [16,17]. In essence, most guidelines from LMICs were largely adopted or adapted from existing HIC guidelines or international and regional organisation guidelines. However, considerations for resource availability, contextual factors, logistical issues and general feasibility are often not made hence affecting implementability of guidelines in these areas. The paucity of locally derived evidence, poorly designed clinical studies, and low weight of evidence [17] have been identified as possible bottlenecks for developing recommendations that are contextually relevant.

### Strengths and limitations of the study

This is the first study to compare specific guideline recommendations for the frequency of monitoring and treatment targets among patients with T2DM, HIV/AIDS or hypertension in a range of countries. Despite its achievements, this study is not without limitations. First, the study did not make use of the instrument that is considered the gold standard for practice guideline evaluation, the Appraisal of Guidelines for Research and Evaluation (AGREE II) [59]. This is because the aim of this study was not to evaluate the quality of the guidelines as a whole, but to compare specific recommendations between countries and to identify the evidence guiding the adoption of the recommendation. Second, the focus on just monitoring frequency and treatment targets can be seen as a weakness as chronic disease management entails more than just monitoring and treatment targets. Third, the country selection procedure was not exhaustive as it was based on previous studies. We, however, believe that the aim of our study is still met, and the analysis is not altered greatly by the countries not selected. Lastly, monitoring often includes aspects of routine health care and/or routine screening for other conditions which may bring indirect benefit to individuals and populations. This was not accounted for in this study.

## CONCLUSIONS AND FUTURE RESEARCH

While clinical practice guidelines seek to address the clear medical need for guidance in the management of chronic conditions, current recommendations for the frequency of monitoring and targets after initiating treatment of T2DM, HIV or hypertension may not meet the needs of local populations at the national or provincial level in LMICs because they are usually drawn from guidelines developed and based on studies carried out in HIC. It might therefore be necessary to generate LMIC-specific fine tuning of recommendations in order to meet the chronic care needs of these populations with an understanding of the socioeconomic context. To generate evidence, in the absence of well designed, adequately powered, randomised, and nonrandomised trials in LMICs, it might be worthwhile to investigate the increased use of simulation studies. It may also be necessary to develop studies that validate current recommendations in LMICs, especially with regards to the frequency of monitoring.

## Additional material


Online Supplementary Document

## References

[1] Checkley W Ghannem H Irazola V Kimaiyo S Levitt NS Miranda JJ Management of NCD in low- and middle-income countries. Glob Heart. 2014;9:431-43. 10.1016/j.gheart.2014.11.003 25592798 PMC4299752

[2] Lall D Engel N Devadasan N Horstman K Criel B Models of care for chronic conditions in low/middle-income countries: a ‘best fit’ framework synthesis. BMJ Glob Health. 2018;3:e001077. 10.1136/bmjgh-2018-001077 30687524 PMC6326308

[3] Fishman P Von Korff M Lozano P Hecht J Chronic care costs in managed care. Health Aff (Millwood). 1997;16:239-47. 10.1377/hlthaff.16.3.239 9141341

[4] Partnership for Solutions. Chronic Conditions: Making the Case for Ongoing Care. Baltimore, MD: Johns Hopkins University; 2002.

[5] Otieno P, Asiki G. Making Universal Health Coverage Effective in Low- and Middle-Income Countries: A Blueprint for Health Sector Reforms, In: Umar Bacha & Urska Rozman & Sonja Sostar Turk (ed.), Healthcare Access - Regional Overviews, IntechOpen. 2020.

[6] Kredo T Abrams A Young T Louw Q Volmink J Daniels K Primary care clinical practice guidelines in South Africa: qualitative study exploring perspectives of national stakeholders. BMC Health Serv Res. 2017;17:608. 10.1186/s12913-017-2546-z 28851365 PMC5575947

[7] Woolf SH Grol R Hutchinson A Eccles M Grimshaw J Clinical guidelines: potential benefits, limitations, and harms of clinical guidelines. BMJ. 1999;318:527-30. 10.1136/bmj.318.7182.527 10024268 PMC1114973

[8] Fischer F Lange K Klose K Greiner W Kraemer A Barriers and Strategies in Guideline Implementation-A Scoping Review. Healthcare (Basel). 2016;4:36. 10.3390/healthcare4030036 27417624 PMC5041037

[9] Olayemi E Asare EV Benneh-Akwasi Kuma AA Guidelines in lower-middle income countries. Br J Haematol. 2017;177:846-54. 10.1111/bjh.14583 28295193

[10] Keiffer MR Utilization of clinical practice guidelines: barriers and facilitators. Nurs Clin North Am. 2015;50:327-45. 10.1016/j.cnur.2015.03.007 25999074

[11] Glasziou P Irwig L Mant D Monitoring in chronic disease: a rational approach. BMJ. 2005;330:644-8. 10.1136/bmj.330.7492.644 15774996 PMC554914

[12] Home P Haddad J Latif ZA Soewondo P Benabbas Y Litwak L Comparison of National/Regional Diabetes Guidelines for the Management of Blood Glucose Control in non-Western Countries. Diabetes Ther. 2013;4:91-102. 10.1007/s13300-013-0022-2 23645286 PMC3687090

[13] Woolf S Schünemann HJ Eccles MP Grimshaw JM Shekelle P Developing clinical practice guidelines: types of evidence and outcomes; values and economics, synthesis, grading, and presentation and deriving recommendations. Implement Sci. 2012;7:61. 10.1186/1748-5908-7-61 22762158 PMC3436711

[14] Dizon JM Machingaidze S Grimmer K To adopt, to adapt, or to contextualise? The big question in clinical practice guideline development. BMC Res Notes. 2016;9:442. 10.1186/s13104-016-2244-7 27623764 PMC5022236

[15] Fervers B Burgers J Haugh M Latreille J Mlika-Cabanne N Paquet L Adaptation of clinical guidelines: literature review and proposition for a framework and procedure. Int J Qual Health Care. 2006;18:167-76. 10.1093/intqhc/mzi108 16766601

[16] Owolabi M Olowoyo P Miranda JJ Akinyemi R Feng W Yaria J Gaps in Hypertension Guidelines in Low- and Middle-Income Versus High-Income Countries: A Systematic Review. Hypertension. 2016;68:1328-37. 10.1161/HYPERTENSIONAHA.116.08290 27698059 PMC5159303

[17] Owolabi MO Yaria JO Daivadanam M Makanjuola AI Parker G Oldenburg B Gaps in Guidelines for the Management of Diabetes in Low- and Middle-Income Versus High-Income Countries-A Systematic Review. Diabetes Care. 2018;41:1097-105. 10.2337/dc17-1795 29678866 PMC5911785

[18] Hajat C Stein E The global burden of multiple chronic conditions: A narrative review. Prev Med Rep. 2018;12:284-93. 10.1016/j.pmedr.2018.10.008 30406006 PMC6214883

[19] Lall D Engel N Devadasan N Horstman K Criel B Models of care for chronic conditions in low/middle-income countries: a ‘best fit’ framework synthesis. BMJ Glob Health. 2018;3:e001077. 10.1136/bmjgh-2018-001077 30687524 PMC6326308

[20] Chhoun P Ngin C Tuot S Pal K Steel M Dionisio J Non-communicable diseases and related risk behaviors among men and women living with HIV in Cambodia: findings from a cross-sectional study. Int J Equity Health. 2017;16:125. 10.1186/s12939-017-0622-y 28705242 PMC5513209

[21] Smit M Brinkman K Geerlings S Smit C Thyagarajan K Sighem AV Future challenges for clinical care of an ageing population infected with HIV: a modelling study [published correction appears in Lancet Infect Dis. 2015 Sep;15(9):998]. Lancet Infect Dis. 2015;15:810-8. 10.1016/S1473-3099(15)00056-0 26070969 PMC4528076

[22] Islam SM Purnat TD Phuong NT Mwingira U Schacht K Fröschl G Non-communicable diseases (NCDs) in developing countries: a symposium report. Global Health. 2014;10:81. 10.1186/s12992-014-0081-9 25498459 PMC4267750

[23] International Diabetes Federation Clinical Guidelines Task Force. Global Guidelines for Type 2 Diabetes, 2017.

[24] Campbell NR Lemogoum D Hypertension in sub-Saharan Africa: a massive and increasing health disaster awaiting solution. Cardiovasc J Afr. 2015;26:152-4. 26407216 PMC4683293

[25] The World Bank. World Bank Country and Lending Groups (2020). Available: https://datahelpdesk.worldbank.org/knowledgebase/articles/906519-world-bank-country-and-lending-groups. Accessed: 30 June 2020.

[26] Rosenfeld RM Shiffman RN Clinical practice guideline development manual: a quality-driven approach for translating evidence into action. Otolaryngol Head Neck Surg. 2009;140(6 Suppl 1):S1-43. 10.1016/j.otohns.2009.04.015 19464525 PMC2851142

[27] Gerstein HC Miller ME Byington RP Goff DC Jr Bigger JT Buse JB Action to Control Cardiovascular Risk in Diabetes Study Group Effects of intensive glucose lowering in type 2 diabetes. N Engl J Med. 2008;358:2545-59. 10.1056/NEJMoa0802743 18539917 PMC4551392

[28] Patel A MacMahon S Chalmers J Neal B Billot L Woodward M ADVANCE Collaborative Group Intensive blood glucose control and vascular outcomes in patients with type 2 diabetes. N Engl J Med. 2008;358:2560-72. 10.1056/NEJMoa0802987 18539916

[29] UK Prospective Diabetes Study (UKPDS) Group Intensive blood-glucose control with sulphonylureas or insulin compared with conventional treatment and risk of complications in patients with type 2 diabetes (UKPDS 33). Lancet. 1998;352:837-53. 10.1016/S0140-6736(98)07019-6 9742976

[30] Ohkubo Y Kishikawa H Araki E Miyata T Isami S Motoyoshi S Intensive insulin therapy prevents the progression of diabetic microvascular complications in Japanese patients with non-insulin-dependent diabetes mellitus: a randomized prospective 6-year study. Diabetes Res Clin Pract. 1995;28:103-17. 10.1016/0168-8227(95)01064-K 7587918

[31] Qaseem A Wilt TJ Kansagara D Horwitch C Barry MJ Forciea MA Clinical Guidelines Committee of the American College of Physicians Hemoglobin A1c Targets for Glycemic Control With Pharmacologic Therapy for Nonpregnant Adults With Type 2 Diabetes Mellitus: A Guidance Statement Update From the American College of Physicians. Ann Intern Med. 2018;168:569-76. 10.7326/M17-0939 29507945

[32] Diabetes Canada Clinical Practice Guidelines Expert Committee Diabetes Canada 2018 Clinical Practice Guidelines for the Prevention and Management of Diabetes in Canada. Can J Diabetes. 2018;42 Suppl 1:S1-325.29650079

[33] Whelton PK Carey RM Aronow WS Casey DE Jr Collins KJ Dennison Himmelfarb C 2017 ACC/AHA/AAPA/ABC/ACPM/AGS/APhA/ASH/ASPC/NMA/PCNA Guideline for the Prevention, Detection, Evaluation, and Management of High Blood Pressure in Adults: Executive Summary: A Report of the American College of Cardiology/American Heart Association Task Force on Clinical Practice Guidelines. Hypertension. 2018;71:1269-24. 10.1161/HYP.0000000000000066 29133354

[34] SPRINT Research Group Wright JT Jr Williamson JD Whelton PK Snyder JK Sink KM A Randomized Trial of Intensive versus Standard Blood-Pressure Control. N Engl J Med. 2015;373:2103-16. 10.1056/NEJMoa1511939 26551272 PMC4689591

[35] Poulter NR Castillo R Charchar FJ Schlaich MP Schutte AE Tomaszewski M Are the American Heart Association/American College of Cardiology High Blood Pressure Guidelines fit for global purpose?: Thoughts from the International Society of Hypertension. Hypertension. 2018;72:260-2. 10.1161/HYPERTENSIONAHA.118.11452 29967044

[36] Rayner B Jones E Veriava Y Seedat YK South African Hypertension Society commentary on the American College of Cardiology/American Heart Association hypertension guidelines. Cardiovasc J Afr. 2019;30:184-7. 10.5830/CVJA-2019-025 31140549

[37] Muntner P Carey RM Gidding S Jones DW Taler SJ Wright JT Jr Potential US Population Impact of the 2017 ACC/AHA High Blood Pressure Guideline. Circulation. 2018;137:109-18. 10.1161/CIRCULATIONAHA.117.032582 29133599 PMC5873602

[38] Wang Z Hao G Wang X Chen Z Zhang L Zhang Z China hypertension survey investigators. Clinical outcomes and economic impact of the 2017 ACC/AHA guidelines on hypertension in China. J Clin Hypertens (Greenwich). 2019;21:1212-20. 10.1111/jch.13609 31267666 PMC8030413

[39] World Health Organization. Consolidated guidelines on the use of antiretroviral drugs for treating and preventing HIV infection. Geneva: World Health Organization;2016. 27466667

[40] Loutfy MR Wu W Letchumanan M Bondy L Antoniou T Margolese S Systematic review of HIV transmission between heterosexual serodiscordant couples where the HIV-positive partner is fully suppressed on antiretroviral therapy. PLoS One. 2013;8:e55747. 10.1371/journal.pone.0055747 23418455 PMC3572113

[41] Opportunistic Infections Project Team of the Collaboration of Observational HIV Epidemiological Research in Europe (COHERE) Mocroft A Reiss P Kirk O Mussini C Girardi E Is it safe to discontinue primary Pneumocystis jiroveci pneumonia prophylaxis in patients with virologically suppressed HIV infection and a CD4 cell count <200 cells/microL? Clin Infect Dis. 2010;51:611-9. 10.1086/655761 20645862

[42] Ford N Stinson K Gale H Mills E Stevens W Pérez González M CD4 changes among virologically suppressed patients on antiretroviral therapy: a systematic review and meta-analysis. J Int AIDS Soc. 2015;18:20061. 10.7448/IAS.18.1.20061 26257204 PMC4530137

[43] Elwenspoek MMC Patel R Watson JC Whiting P Are guidelines for monitoring chronic disease in primary care evidence based? BMJ. 2019;365:l2319. 10.1136/bmj.l2319 31196976

[44] Fu C Ji L Wang W Luan R Chen W Zhan S Frequency of glycated hemoglobin monitoring was inversely associated with glycemic control of patients with Type 2 diabetes mellitus. J Endocrinol Invest. 2012;35:269-73. 21606668 10.3275/7743

[45] Driskell OJ Holland D Waldron JL Ford C Scargill JJ Heald A Reduced testing frequency for glycated hemoglobin, HbA1c, is associated with deteriorating diabetes control. Diabetes Care. 2014;37:2731-7. 10.2337/dc14-0297 25249670

[46] Canadian Agency for Drugs and Technologies in Health. HbA1c Testing Frequency: A Review of the Clinical Evidence and Guidelines. Ottawa: Canadian Agency for Drugs and Technologies in Health; 2014. 25411681

[47] Oke JL Stevens RJ Gaitskell K Farmer AJ Establishing an evidence base for frequency of monitoring glycated haemoglobin levels in patients with Type 2 diabetes: projections of effectiveness from a regression model. Diabet Med. 2012;29:266-71. 10.1111/j.1464-5491.2011.03412.x 21838767

[48] Birtwhistle RV Godwin MS Delva MD Casson RI Lam M MacDonald SE Randomised equivalence trial comparing three month and six month follow up of patients with hypertension by family practitioners. BMJ. 2004;328:204. 10.1136/bmj.37967.374063.EE 14726370 PMC318487

[49] Keenan K Hayen A Neal BC Irwig L Long term monitoring in patients receiving treatment to lower blood pressure: analysis of data from placebo controlled randomised controlled trial. BMJ. 2009;338:b1492. 10.1136/bmj.b1492 19406886 PMC2675695

[50] Takahashi O Glasziou PP Perera R Shimbo T Fukui T Blood pressure re-screening for healthy adults: what is the best measure and interval? J Hum Hypertens. 2012;26:540-6. 10.1038/jhh.2011.72 21814284

[51] National Heart Foundation of Australia. Guideline for the diagnosis and management of hypertension in adults – 2016. Melbourne: National Heart Foundation of Australia; 2016.

[52] Calmy A Ford N Hirschel B Reynolds SJ Lynen L Goemaere E HIV viral load monitoring in resource-limited regions: optional or necessary? Clin Infect Dis. 2007;44:128-34. 10.1086/510073 17143828

[53] Reekie J Mocroft A Sambatakou H Machala L Chiesi A van Lunzen J Does less frequent routine monitoring of patients on a stable, fully suppressed cART regimen lead to an increased risk of treatment failure? AIDS. 2008;22:2381-90. 10.1097/QAD.0b013e328317a6eb 18981778

[54] Caniglia EC Sabin C Robins JM Logan R Cain LE Abgrall S When to Monitor CD4 Cell Count and HIV RNA to Reduce Mortality and AIDS-Defining Illness in Virologically Suppressed HIV-Positive Persons on Antiretroviral Therapy in High-Income Countries: A Prospective Observational Study. J Acquir Immune Defic Syndr. 2016;72:214-21. 10.1097/QAI.0000000000000956 26895294 PMC4866894

[55] Romih V Zidovec Lepej S Gedike K Lukas D Begovac J. Frequency of HIV-1 viral load monitoring of patients initially successfully treated with combination antiretroviral therapy. PloS One. 2010;5:e15051. 10.1371/journal.pone.0015051 21124844 PMC2991345

[56] Rafiee M Kariminia A Wright S Mills G Woolley I Smith D Reducing Viral Load Measurements to Once a Year in Patients on Stable, Virologically Suppressive Cart Regimen: Findings from the Australian HIV Observational Database. J AIDS Clin Res. 2014;5:383. 26618053 10.4172/2155-6113.1000383 PMC4662571

[57] Guyatt G Oxman AD Akl EA Kunz R Vist G Brozek J GRADE guidelines: 1. Introduction-GRADE evidence profiles and summary of findings tables. J Clin Epidemiol. 2011;64:383-94. 10.1016/j.jclinepi.2010.04.026 21195583

[58] Ebell MH Siwek J Weiss BD Woolf SH Susman J Ewigman B Strength of Recommendation Taxonomy (SORT): A patient-Centered Approach to Grading Evidence in the Medical Literature. J Am Board Fam Pract. 2004;17:59-67. 10.3122/jabfm.17.1.59 15014055

[59] Brouwers MC Kho ME Browman GP Burgers JS Cluzeau F Feder G AGREE II: advancing guideline development, reporting and evaluation in health care. CMAJ. 2010;182:E839-42. 10.1503/cmaj.090449 20603348 PMC3001530

[60] Attia A Adaptation of international evidence based clinical practice guidelines: The ADAPTE process. Middle East Fertil Soc J. 2013;18:123-6. 10.1016/j.mefs.2013.03.002

