# Supplementary material for: A comparative analysis and review of how national guidelines for chronic disease monitoring are made in low- and middle-income compared to high-income countries
Source: J Glob Health. 2021 Sep 4;11:04055. doi: 10.7189/jogh.11.04055 (PMC8442582; doi:10.7189/jogh.11.04055)
Supplement: Online Supplementary Document [file jogh-11-04055-s001.pdf]

Title: "A comparative analysis and review of how national guidelines for chronic disease monitoring are made in low- and middle-income compared to high-income countries."

Authors: Elton Mukonda[1], Maia Lesosky[1]

Affiliation:

[1]Division of Epidemiology & Biostatistics, School of Public Health & Family Medicine, University ofCape Town, Cape Town, South Africa

Supplementary material

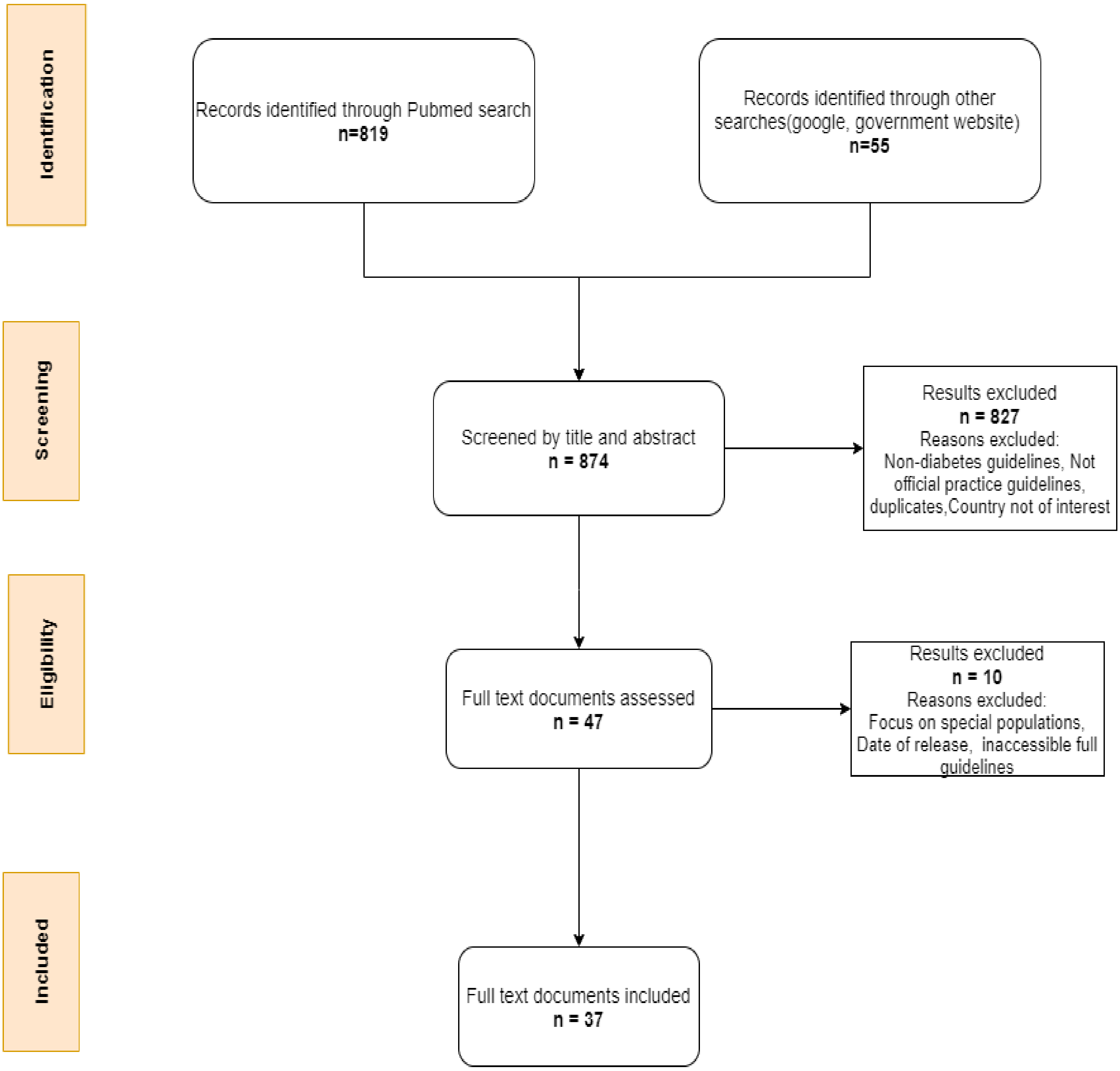

Figure S1: PRISMA Flow Chart of the Diabetes guidelines included and excluded from the analysis

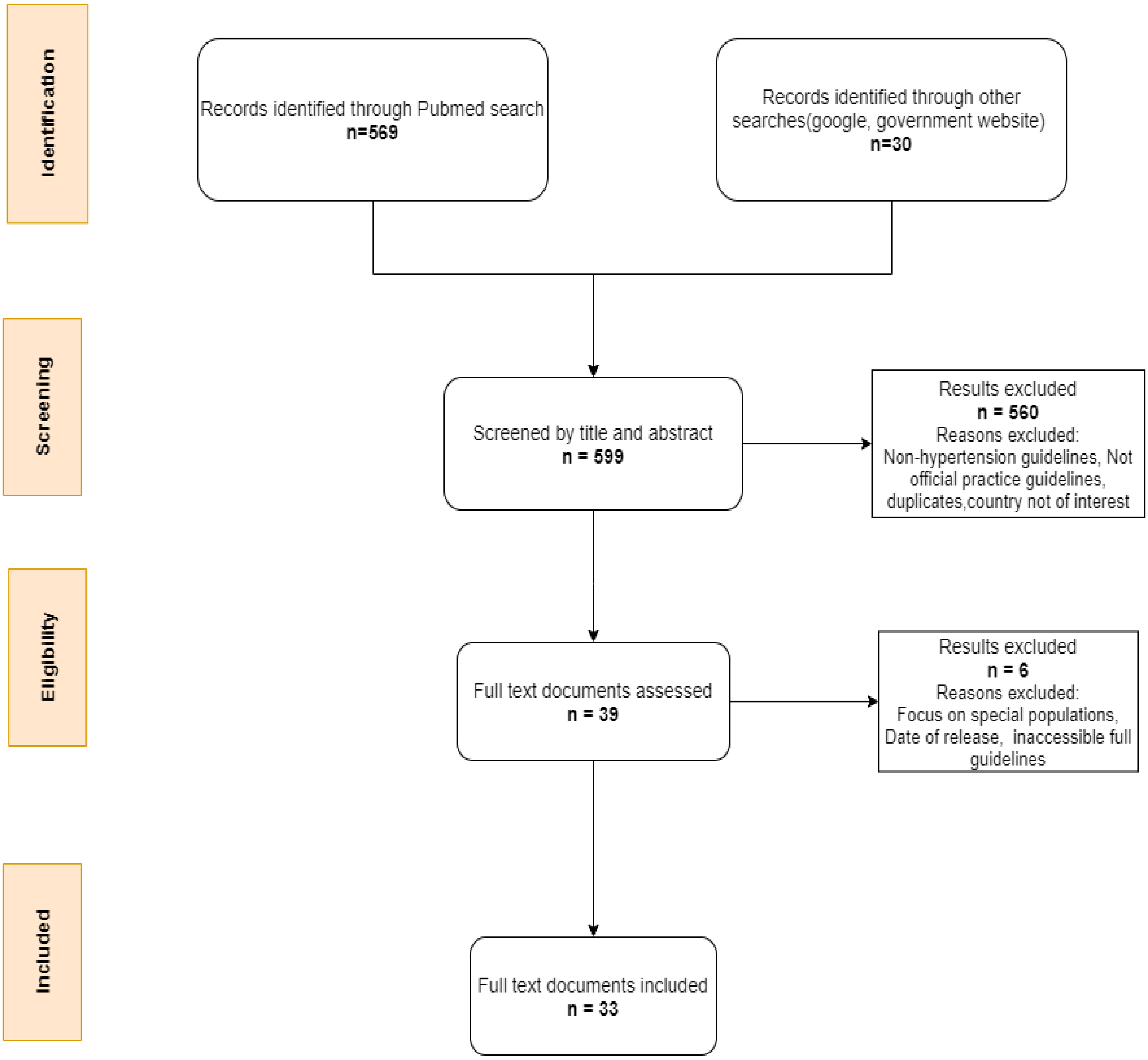

Figure S2: PRISMA Flow Chart of the Hypertension guidelines included and excluded from the analysis

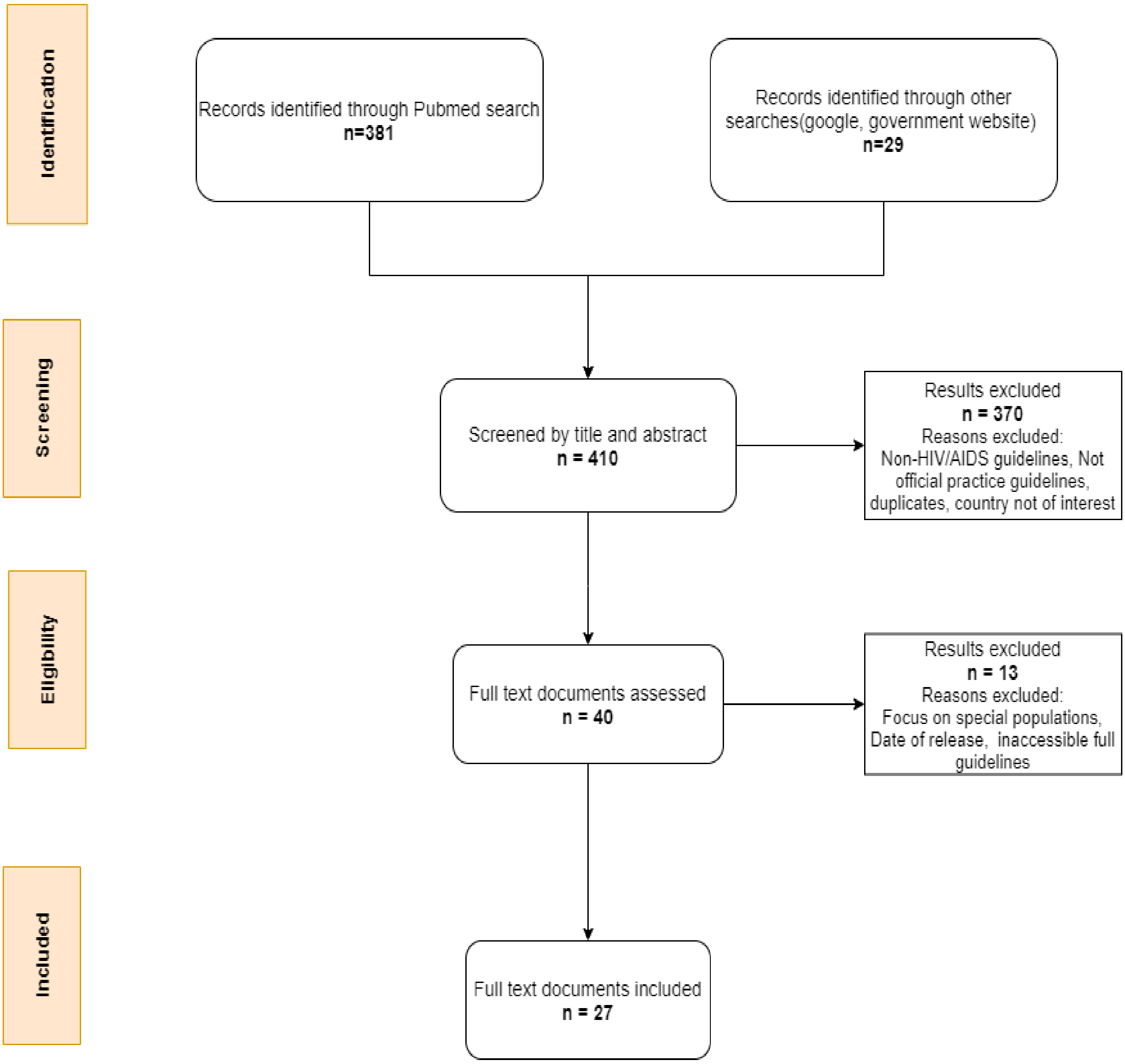

Figure S3: PRISMA Flow Chart of the HIV/AIDS guidelines included and excluded from the analysis

Table S1: Guidelines and targets for monitoring HbA1c in LMICs and HICs among Type 2 Diabetes patients

Country	Classification	Document title	Guideline developer	Year	Control Target	Monitoring frequency: Newly diagnosed patients	Monitoring frequency: Stable patients	Evidence / Strength of Recommendations (Treatment targets)	Evidence / Strength of Recommendations (Frequency of monitoring)
Australia [1]	HIC	General practice management of type 2 diabetes	The Royal Australian College of General Practitioners/ Diabetes Australia	2020	≤7.0%	3 – 6 monthly	3 – 6 monthly	Yes	Yes
Bahrain [2]	HIC	Guideline for Management of Type 2 Diabetes Mellitus,	Ministry of Health	2016	≤7.0%	2 – 6 monthly	2 – 6 monthly	No	No
^ Bermuda [3]	HIC	Guidelines for Diabetes Care in Bermuda	Department of Health	2009	≤7.0%	3-monthly	6-monthly	No	No
Brazil [4]	Upper-MIC	Diretrizes da Sociedade Brasileira de Diabetes 2019-2020	Brazilian Diabetes Society	2019	≤7.0%	Not provided	6-monthly	Yes	Yes
Canada [5]	HIC	Clinical Practice Guidelines for the Prevention and Management of Diabetes in Canada	Diabetes Canada (formerly Canadian Diabetes Association)	2018	≤7.0%	3-monthly	6-monthly	Yes	Yes
China [6]	Upper-MIC	Standards of medical care for type 2 diabetes in China	Chinese Diabetes Society (CDS)	2019	≤7.0%	3-monthly	6-monthly	Yes	No
Colombia [7]	Upper-MIC	Clinical practice guideline for the prevention, early detection, diagnosis, management and follow up of type 2	Ministry of Health and Social Welfare	2016	≤7.0%	3 – 6 monthly	3 – 6 monthly	Yes	No

		diabetes mellitus in adults							
Ethiopia [8]	LIC	Standard Treatment Guidelines For General Hospitals	Ethiopian Food, Medicine and Healthcare Administration and Control Authority (EFMHACA)	2014	≤7.0%	Not provided	Not provided	No	No
Europe [9]	Regional	ESC Guidelines on diabetes, pre-diabetes, and cardiovascular diseases developed in collaboration with the EASD	European Society of Cardiology (ESC) and the European Association for the Study of Diabetes (EASD)	2019	≤7.0%	Not provided	Not provided	Yes	No
Fiji [10]	Upper-MIC	Diabetes Management Guidelines	Ministry of Health	2012	≤7.0%	Not provided	Not provided	No	No
Ghana [11]	Lower-MIC	Standard Treatment Guidelines	Ministry of Health	2017	≤7.0%	6-monthly	6-monthly	No	No
Greece [12]	HIC	Guidelines for the management of the diabetic patient	Hellenic Diabetes Association	2018	≤7.0%	3-monthly	6-monthly	Yes	Yes
Hong Kong [13]	HIC	Hong Kong Reference Framework for Diabetes Care for Adults in Primary Care Settings	Food and Health Bureau	2018	≤7.0%	3-monthly	6-monthly	Yes	Yes
India [14]	Lower-MIC	Guidelines For Management of Type 2 Diabetes 2018	Indian Council of Medical Research (ICMR)	2018	≤7.0%	3-monthly	6 to 12-monthly	No	No
International [15,16]	International	Global Guidelines for Type 2 Diabetes,	International Diabetes Federation (IDF)	2017, 2012	≤7.0%	2 – 6 monthly	2 – 6 monthly	No	No
Kenya [17]	Lower-MIC	National Clinical Guidelines for	Ministry of Health	2010	≤7.0%	6-monthly	6-monthly	No	No

		Management of Diabetes Mellitus							
Korea Republic [18]	HIC	Clinical Practice Guidelines for Type 2 Diabetes Mellitus in Korea	Korean Diabetes Association	2019	≤6.5%	Not provided	Not provided	Yes	No
Latin America [19,20]	Regional	Guidelines on the Diagnosis, Control and Treatment of Type 2 Diabetes Mellitus with Evidence-Based Medicine	Latin American Diabetes Association, ALAD	2019, 2010	≤7.0%	3 – 4 monthly	3 – 4 monthly	No	No
Libya [21]	Upper-MIC	LIBYAN DIABETES CARE GUIDELINES (2010-2012).	Libyan Ministry of Health, Libyan Board of Medical Specialization, Libyan Association for Diabetes and Endocrinology	2010	6.5-7.0	3 – 6 monthly	3 – 6 monthly	No	No
Malaysia [22]	Upper-MIC	Clinical Practice Guidelines (CPG): Management of Type 2 Diabetes Mellitus (T2DM)	Ministry of Health/Malaysian Endocrine and Metabolic Society (MEMS)	2015	≤6.5%	3-monthly	6-monthly	Yes	No
Mexico [23]	Upper-MIC	Official Mexican Standard PROY-NOM-015-SSA2-2018, For the prevention, detection, diagnosis, treatment and control of Diabetes Mellitus	Ministry of Health	2017	≤7%	Not provided	Not provided	No	No
New Zealand [24]	HIC	Guidance on the Management of type 2 diabetes 2011	New Zealand Guidelines Group, Ministry of Health	2011	6.7–7.2%	3 – 6 monthly	3 – 6 monthly	No	No

Nigeria [25]	Lower-MIC	Guidelines for Diabetes Management In Nigeria	Diabetes Association of Nigeria (DAN)	2013	≤6.5%	3-monthly	6-monthly	No	No
Peru [26]	Upper-MIC	Guía de práctica clínica para el Diagnóstico, Tratamiento y Control de la Diabetes Mellitus tipo 2 en el Primer Nivel de Atención.	Ministry of Health (Ministerio de Salud del)	2016	≤7.0%	Not provided	6-monthly	Yes	Yes
the Philippines [27]	Lower-MIC	Philippine Practice Guidelines for the Diagnosis and Management of Diabetes.	Unite for Diabetes Philippines	2010	≤7.0%	3 – 6 monthly	3 – 6 monthly	Yes	Yes
Scotland [28]	HIC	Pharmacological management of glycaemic control in people with type 2 diabetes.	Scottish Intercollegiate Guidelines Network (SIGN)	2017	≤7.0%	Not provided	Not provided	Yes	No
Singapore [29] *	HIC	Clinical practice guidelines Diabetes Mellitus	Ministry of Health	2014	≤7.0%	3 – 4 monthly	6-monthly	Yes	Yes
South Africa [30]	Upper-MIC	The 2017 SEMDSA Guideline for the Management of Type 2 Diabetes	Society for Endocrinology, Metabolism and Diabetes of South Africa (SEMDSA)	2017	≤7.0%	3-monthly	6-monthly	Yes	Yes
Spain [31]	HIC	Spanish Diabetes Society (SED) recommendations for the pharmacologic treatment of hyperglycemia in type 2 diabetes	Spanish Diabetes Society (SED)	2018	≤6.5%	Not provided	Not provided	Yes	No
Sri Lanka [32]	Lower-MIC	Diabetes Mellitus: Glucose Control	SL College of Endocrinologists	2013	≤7.0%	3-monthly	6-monthly	No	No
Eswatini (formerly Swaziland	Lower-MIC	Standard Treatment Guidelines and Essential Medicines	Ministry of Health	2012	≤6.5%	6-monthly	Annually	No	No

) [33]		List of Common Medical Conditions							
Sweden [34]	HIC	National Guidelines for Diabetes Care.	Swedish National Board of Health and Welfare	2015	≤6.9%	Not provided	Not provided	Yes	No
Tanzania [35]	LIC	Standard Treatment Guidelines (STG) and National Essential Medicines List	Ministry of Health,	2017	≤6.5%	6-monthly	6-monthly	No	No
Turkey [36]	Upper-MIC	Clinical Practice Guideline for Diagnosis, Treatment and Follow-up of Diabetes Mellitus and Its Complications - 2019	SOCIETY of ENDOCRINOLOGY and METABOLISM of TURKEY	2019	≤7.0%	3-monthly	6-monthly	Yes	No
U.S. [37]	HIC	Standards of Medical Care in Diabetes	American Diabetes Association (ADA)	2020	≤7.0%	3-monthly	6-monthly	Yes	Yes
Uganda [38]	LIC	Clinical Practice Guidelines	Ministry of Health	2016	≤7.0%	Not provided	Not provided	No	No
United Kingdom [39]	HIC	Type 2 diabetes: The management of type 2 diabetes,	National Institute for Health and Care Excellence (NICE)	2015	≤6.5%	3 – 6 monthly	6-monthly	Yes	Yes

- *Withdrawn

Table S2: Guidelines and targets for monitoring viral load in LMICs and HICs among HIV patients on ART

Country	Classification	Document title	Guideline developer	Year	Control Target	Monitoring frequency: Newly diagnosed patients	Monitoring frequency: Stable patients	Evidence / Strength of Recommendations (Treatment targets)	Evidence / Strength of Recommendations (Frequency of monitoring)
Australia [40]	HIC	Antiretroviral guidelines: US DHHS guidelines with Australian commentary. 2019	Australasian Society for HIV, Viral Hepatitis and Sexual Health Medicine (ASHM)	2019	<200 copies/mL	4 - 6 weekly	3 - 6 monthly	Yes	No
Botswana [41]	Upper-MIC	Handbook of the Botswana 2016 Integrated HIV Clinical Care Guidelines.	Ministry of Health	2016	<400 copies/mL	Not provided	Not provided	No	No
Canada [42]	HIC	THERAPEUTIC GUIDELINES ANTIRETROVIRAL (ARV) TREATMENT OF ADULT HIV INFECTION	British Columbia Centre for Excellence in HIV/AIDS (BC-CfE)	2019	<200 copies/mL	4 - 6 weekly	3 - 6 monthly	Yes	No
China [43]	Upper-MIC	Chinese AIDS Diagnosis and Treatment Guide	AIDS Professional Group, Society of Infectious Diseases, Chinese Medical Association.	2018	<200 copies/mL	Not provided	Annually	Yes	No
Ethiopia [44]	LIC	NATIONAL CONSOLIDATED GUIDELINES FOR COMPREHENSIVE HIV PREVENTION, CARE AND TREATMENT	Federal Ministry of Health	2018	<1000 copies/mL	6-monthly	Annually	No	No
Europe [45]	Regional	EACS Guidelines 2020 version 10.1	European AIDS Clinical Society (EACS)	2020	<200 copies/mL	3 - 6 monthly	3 - 6 monthly	Yes	No

Ghana [46]	Lower-MIC	GUIDELINES FOR ANTIRETROVIRAL THERAPY IN GHANA	National AIDS/STI Control Programme (NACP), Ghana Health Service	2010	<1000 copies/mL	6-monthly	Annually	No	No
India [47]	Lower-MIC	NATIONAL TECHNICAL GUIDELINES ON ANTI RETROVIRAL TREATMENT	Ministry of Health	2018	<1000 copies/mL	6-monthly	Annually	No	No
International [48]	International	Consolidated guidelines on the use of antiretroviral drugs for treating and preventing HIV infection: recommendations for a public health approach	World Health Organisation (WHO)	2016	<1000 copies/mL	6-monthly	Annually	Yes	Yes
Jamaica [49]	Upper-MIC	CLINICAL MANAGEMENT OF HIV DISEASE: Guidelines for Medical Practitioners	Ministry of Health	2017	<1000 copies/mL	6-monthly	6 - 12 monthly	No	No
Kenya [50]	Lower-MIC	Guidelines on Use of Antiretroviral Drugs for Treating and Preventing HIV Infection in Kenya 2018 Edition	Ministry of Health	2018	<1000 copies/mL	6-monthly	Annually	No	No
Korea Republic [51]	HIC	The 2018 Clinical Guidelines for the Diagnosis and Treatment of HIV/AIDS in HIV-Infected Koreans	Korean Society for AIDS	2018	<200 copies/mL	Not provided	24-weekly	Yes	No
Liberia [52]	LIC	. National Standard Therapeutic Guidelines and	Ministry of Health, Standard	2017	<1000 copies/mL	Not provided	Not provided	No	No

		Essential MedicinesList	Treatment Guidelines						
Malawi [53]	LIC	Guidelines for Clinical Managementof HIV in Children and Adults,	Ministry of Health	2016	<1000 copies/mL	6-monthly	2-yearly	No	No
Malaysia [54]	Upper-MIC	Malaysian Consensus Guidelines on Antiretrovira lTherapy 2017	Ministry of Health	2017	<1000 copies/mL	4 - 6 monthly	6 - 12 monthly	No	No
Nigeria [55]	Lower-MIC	Standard Treatment Guideline	Ministry of Health	2016	<1000 copies/mL	6-monthly	Annually	No	No
Singapore [56]	HIC	Recommendations for the Use of Antiretroviral Therapy in Adults Living with HIV in Singapore	Ministry of Health/National Center for Infectious Diseases	2019	<1000 copies/mL	6-monthly	Annually	Yes	Yes
South Africa [57]	Upper-MIC	2019 ART Clinical Guidelines for the Management of HIV in Adults, Pregnancy, Adolescents, Children, Infants and Neonates.	Ministry of Health	2019	<1000 copies/mL	6-monthly	Annually	No	No
Sri Lanka [58]	Lower-MIC	The Guideline Use ofAntiretroviral Drugsfor Treating and Prevention of HIV Infection.	Ministry of Health	2014	<1000 copies/mL	6-monthly	Annually	No	No
Eswatini (formerly Swaziland) [59]	Lower-MIC	Integrated HIV Management Guidelines.	Ministry of Health	2015	<1000 copies/mL	6-monthly	Annually	No	No

Sweden [60]	HIC	Antiretroviral treatment for HIV infection: Swedish recommendation s	Swedish Medical Products Agency and the Swedish Reference Group for Antiviral Therapy (RAV)	2016	<150 copies/mL	3-monthly	3-monthly	Yes	No
Tanzania [35]	LIC	Standard Treatment Guidelines (STG) and National Essential Medicines List TANZANIA MAINLAND (NEMLIT)	Ministry of Health, Standard Treatment Guidelines	2017	<1000 copies/mL	6-monthly	Annually	No	No
U.S. [61]	HIC	Guidelines for the Use of Antiretroviral Agents in Adults andAdolescents with HIV	Department of Health and Human Services (DHHS)	2018	<200 copies/mL	3 - 4 monthly	6-monthly	Yes	Yes
Uganda [38]	LIC	. Uganda Clinical Guidelines 2016 – National Guidelinesfor Management of Common Conditions	Ministry of Health, Clinical Practice Guidelines	2016	<1000 copies/mL	6-monthly	Annually	No	No
United Kingdom [62]	HIC	BHIVA guidelines forthe routine investigation and monitoring of adult HIV-1-positive individuals 2016	British HIV Association (BHIVA)	2019	<200 copies/mL	3-monthly	6 - 12 monthly	Yes	Yes
Zambia [63]	Lower-MIC	Zambia Consolidated Guidelines for Treatment and Prevention of HIVInfection	Ministry of Health	2020	<1000 copies/mL	6-monthly	Annually	No	No

Zimbabwe [64]	LIC	Guidelines for Antiretroviral Therapy for the Prevention and Treatment of HIV in Zimbabwe.	Ministry of Health	2016	<1000 copies/mL	6-monthly	Annually	No	No

Table S3: Guidelines and targets for monitoring blood pressure in LMICs and HICs among Hypertensives

Country	Classification	Document title	Guideline developer	Year	Control Target	Monitoring frequency: Newly diagnosed patients	Monitoring frequency: Stable patients	Evidence / Strength of Recommendations (Treatment targets)	Evidence / Strength of Recommendations (Frequency of monitoring)
Afghanistan [65]	LIC	National Standard Treatment Guidelines for the Primary Level.	Ministry of Public Health, National Standard Treatment Guidelines for the Primary Level.	2013	<140/90 mmHg	Not provided	Not provided	No	No
Argentina [66]	HIC	Principal conclusions of the Argentine Consensus on Arterial Hypertension	Argentine Society of Arterial Hypertension/Argentine Society of Cardiology/Argentine Federation of Cardiology	2018	<140/90 mmHg	Not provided	Not provided	Yes	No
Australia [67]	HIC	Guideline for the diagnosis and management of hypertension in adults – 2016.	National Heart Foundation of Australia	2016	<140/90 mmHg	4 - 6 weekly	3 - 6 monthly	Yes	Yes
*Bahrain [68]	HIC	Hypertension in clinical practice.	Ministry of Health	2011	<140/90 mmHg	Not provided	Not provided	No	No
Bermuda [69]	HIC	Hypertension Guidelines for Bermuda	Ministry of Health	2011	<140/90 mmHg	3 - 6 monthly	3 - 6 monthly	No	No
Botswana [70]	Upper-MIC	Practical Approach to Care Kit (PACK) - Botswana Primary Care Guideline for Adult.	Ministry of Health	2013	<140/90 mmHg	Not provided	6-monthly	No	No
Brazil [71]	Upper-MIC	7th Brazilian Guideline of Arterial Hypertension:	Brazilian Society of Cardiology	2016	<140/90 mmHg	Not provided	Not provided	Yes	No

		Chapter 5 - Therapeutic Decision and Targets							
Canada [72]	HIC	Hypertension Canada's 2018 Guidelines for Diagnosis, Risk Assessment, Prevention, and Treatment of Hypertension in Adults and Children	Hypertension Canada	2018	<140/90 mmHg	Not provided	Not provided	Yes	No
China [73]	Upper-MIC	2018 Chinese Guidelines for Prevention and Treatment of Hypertension	Chinese Hypertension League (CHL)	2018	<140/90 mmHg	2 - 3 monthly	Not provided	Yes	No
Ethiopia [8]	LIC	NATIONAL CONSOLIDATED GUIDELINES FOR COMPREHENSIV E HIV PREVENTION, CARE AND TREATMENT	Ethiopian Food, Medicine and Healthcare Administration and Control Authority (EFMHACA), Standard Treatment Guidelines	2014	<140/90 mmHg	Not provided	Not provided	No	No
Europe [74]	Regional	2019 ESC Guidelines on diabetes, pre-diabetes, and cardiovascular diseases developed in collaboration with the EASD	European Society of Cardiology (ESC) and the European Society of Hypertension (ESH)	2018	<140/90 mmHg	Not provided	3 - 6 monthly	Yes	Yes
Fiji [75]	Upper-MIC	Cardiovascular Therapeutic Guidelines.	Ministry of Health and Medical Services	2015	<140/90 mmHg	Not provided	Not provided	No	No

Ghana [11]	Lower-MIC	Standard Treatment Guidelines	Ministry of Health,	2017	<140/90 mmHg	Not provided	Not provided	No	No
Hong Kong [76]	HIC	Hong Kong SAR Government. Hong Kong Reference Framework for **Hypertension** Care for Adults in Primary Care Settings	Food and Health Bureau	2018	<140/90 mmHg	Not provided	6 - 12 weekly	Yes	No
India [77]	Lower-MIC	Indian guidelines on hypertension-IV	Association of Physicians of India (API)	2019	<130/80 mmHg	3-monthly	6-monthly	No	No
International [78]	International	2020 International Society of Hypertension Global Hypertension Practice Guidelines.	International Society of Hypertension (ISH)	2020	<140/90 mmHg	Not provided	Not provided	Yes	No
Jamaica [79]	Upper-MIC	GUIDELINES FOR THE MANAGEMENT OF HYPERTENSION	Ministry of Health	2014	<140/90 mmHg	Monthly	3 - 6 monthly	No	No
Kenya [80]	Lower-MIC	KENYA NATIONAL GUIDELINES FOR CARDIOVASCULAR DISEASES MANAGEMENT	Ministry of Health	2018	<140/90 mmHg	Monthly	4 - 6 monthly	No	No
Korea Republic [81]	HIC	Guidelines for the management of hypertension: part II-diagnosis	Korean society of hypertension	2018	<140/90 mmHg	Monthly	3 - 6 monthly	Yes	No

		and treatment of hypertension							
Latin America [82]	Regional	Guidelines on the management of arterial hypertension and related comorbidities in Latin America	Latin American Society of Hypertension	2017	<140/90 mmHg	Not provided	Not provided	No	No
Liberia [52]	LIC	National Standard Therapeutic Guidelines and Essential Medicines List	Ministry of Health, Standard Treatment Guidelines	2017	<140/90 mmHg	Not provided	Not provided	No	No
Malawi [83]	LIC	Malawi Standard Treatment Guidelines (MSTG)	Ministry of Health, Standard Treatment Guidelines	2015	<140/90 mmHg	Not provided	Not provided	No	No
Malaysia [84]	Upper-MIC	Clinical practice guidelines: management of hypertension	Ministry of Health Malaysia/Malaysian Society of Hypertension	2018	<140/90 mmHg	Not provided	3 - 6 monthly	Yes	No
Nigeria [55]	Lower-MIC	Standard Treatment Guidelines	Ministry of Health,	2016	<140/90 mmHg	Not provided	Not provided	No	No
Scotland [85]	HIC	SIGN 149 - Risk estimation and the prevention of cardiovascular disease. A national clinical guideline	Scottish Intercollegiate Guidelines Network	2017	<140/90 mmHg	Not provided	Not provided	Yes	No
Singapore [86]	HIC	Clinical practice guidelines Hypertension.	Ministry of Health	2017	<140/90 mmHg	3 - 6 monthly	6 - 12 monthly	Yes	Yes
South Africa [87]	Upper-MIC	South African hypertension	Southern African Hypertension Society (SAHS)	2014,2011	<140/90 mmHg	2-monthly	3 - 6 monthly	No	No

		practice guideline 2014							
Sri Lanka [88]	Lower-MIC	CLINICAL PRACTICE GUIDELINES: HYPERTENSION MANAGEMENT GUIDELINES	CCP - Ceylon College of Physicians	2016	<140/90 mmHg	3-monthly	3-monthly	No	No
Eswatini (formerly Swaziland) [33]	Lower-MIC	Standard Treatment Guidelines and Essential Medicines List of Common Medical Conditions	Ministry of Health,	2012	<140/90 mmHg	Monthly	4 - 6 monthly	No	No
Tanzania [35]	LIC	Standard Treatment Guidelines (STG) and National Essential Medicines List	Ministry of Health,	2017	<140/90 mmHg	1 - 3 monthly	6-monthly	No	No
U.S. [89]	HIC	ACC/AHA/AAPA/ ABC/ACPM/AGS/ APhA/ASH/ASPC /NMA/PCNA guideline for the prevention, detection, evaluation, and management of high blood pressure in adults	American College of Cardiology (ACC) and American Heart Association (AHA)	2018	<130/80 mmHg	Monthly	3 - 6 monthly	Yes	Yes
Uganda [38]	LIC	Uganda Clinical Guidelines 2016 – National Guidelines for Management of	Ministry of Health, Clinical Practice Guidelines	2016	<140/90 mmHg	Not provided	Not provided	No	No

		Common Conditions							
United Kingdom [90]	HIC	Hypertension in adults: diagnosis and management (NG136)	National Institute for Health and Care Excellence (NICE)	2019	<140/90 mmHg	Annually	Annually	Yes	Yes

Table S4: Search strategy for Diabetes guidelines

Search number	Query	Filters	Results
1	"diabete"[All Fields] OR "diabetes mellitus"[MeSH Terms] OR ("diabetes"[All Fields] AND "mellitus"[All Fields]) OR "diabetes mellitus"[All Fields] OR "diabetes"[All Fields] OR "diabetes insipidus"[MeSH Terms] OR ("diabetes"[All Fields] AND "insipidus"[All Fields]) OR "diabetes insipidus"[All Fields] OR "diabetic"[All Fields] OR "diabetics"[All Fields] OR "diabets"[All Fields]		814,997
2	"guideline"[Publication Type] OR "guidelines as topic"[MeSH Terms] OR "guidelines"[All Fields] OR "consensual"[All Fields] OR "consensually"[All Fields] OR "consensus"[MeSH Terms] OR "consensus"[All Fields] OR "recommend"[All Fields] OR "recommendable"[All Fields] OR "recommendation"[All Fields] OR "recommendation s"[All Fields] OR "recommendations"[All Fields] OR "recommended"[All Fields] OR "recommending"[All Fields] OR "recommends"[All Fields]		1,217,224
3	1 AND 2		42,309
4	1 AND 2	from 2010 - 2020	26,109
5	1 AND 2	Government Publication, Practice Guideline, from 2010 - 2020	819

Table S5: Search strategy for hypertension guidelines

Search number	Query	Filters	Results
1	"hypertense"[All Fields] OR "hypertension"[MeSH Terms] OR "hypertension"[All Fields] OR "hypertension s"[All Fields] OR "hypertensions"[All Fields] OR "hypertensive"[All Fields] OR "hypertensive s"[All Fields] OR "hypertensives"[All Fields]		561,827
2	"guideline"[Publication Type] OR "guidelines as topic"[MeSH Terms] OR "guidelines"[All Fields] OR "consensual"[All Fields] OR "consensually"[All Fields] OR "consensus"[MeSH Terms] OR "consensus"[All Fields] OR "recommend"[All Fields] OR "recommendable"[All Fields] OR "recommendation"[All Fields] OR "recommendation s"[All Fields] OR "recommendations"[All Fields] OR "recommended"[All Fields] OR "recommending"[All Fields] OR "recommends"[All Fields]		1,217,224
3	1 AND 2		35,988
4	1 AND 2	from 2010 - 2020	20,837
5	1 AND 2	Government Publication, Practice Guideline, from 2010 - 2020	569

Table S6: Search strategy for HIV/AIDs guidelines

Search number	Query	Filters	Results
1	"acquired immunodeficiency syndrome"[MeSH Terms] OR ("acquired"[All Fields] AND "immunodeficiency"[All Fields] AND "syndrome"[All Fields]) OR "acquired immunodeficiency syndrome"[All Fields] OR "aids"[All Fields] OR ("hiv"[MeSH Terms] OR "hiv"[All Fields])		493,182
2	"guideline"[Publication Type] OR "guidelines as topic"[MeSH Terms] OR "guidelines"[All Fields] OR "consensual"[All Fields] OR "consensually"[All Fields] OR "consensus"[MeSH Terms] OR "consensus"[All Fields] OR "recommend"[All Fields] OR "recommendable"[All Fields] OR "recommendation"[All Fields] OR "recommendation s"[All Fields] OR "recommendations"[All Fields] OR "recommended"[All Fields] OR "recommending"[All Fields] OR "recommends"[All Fields]		1,217,224
3	1 AND 2		36,229
4	1 AND 2	from 2010 - 2020	19,167
5	1 AND 2	Government Publication, Practice Guideline, from 2010 - 2020	381

Diabetes

1. The Royal Australian College of General Practitioners. Management of type 2 diabetes: A handbook for general practice. East Melbourne, Vic: RACGP, 2020. Available from: http://www.racgp.org.au/your-practice/guidelines/diabetes/

2. Ministry of Health Kingdom of Bahrain. Guideline for Management of Type 2 Diabetes Mellitus: Ministry of Health, Kingdom of Bahrain; 2016.

3. Diabetes Task Group. Guidelines for Diabetes Care in Bermuda. Government of Bermuda: Department of Health. 2009

4. Diabetes, Sociedade & Silva Júnior, Wellington. Diretrizes da Sociedade Brasileira de Diabetes 2019-2020. 2019

5. Diabetes Canada Clinical Practice Guidelines Expert Committee. Diabetes Canada 2018 Clinical Practice Guidelines for the Prevention and Management of Diabetes in Canada. Can J Diabetes. 2018;42(Suppl 1):S1-S325.

6. Jia W, Weng J, Zhu D, Ji L, Lu J, Zhou Z, et al On behalf of Chinese Diabetes Society. Standards of medical care for type 2 diabetes in China 2019, Diabetes Metab Res Rev. 2019; 35:e3158. https://doi.org/10.1002/dmrr.3158.

7. Aschner PM, Muñoz OM, Girón D, García OM, Fernández-Ávila DG, Casas LÁ, et al. Clinical practice guideline for the prevention, early detection, diagnosis, management and follow up of type 2 diabetes mellitus in adults. Colomb Med (Cali). 2016 Jun 30;47(2):109-31. PMID: 27546934; PMCID: PMC4975132.

8. Standard Treatment Guidelines for General Hospitals. Ethiopia, Third Edition, 2014. Available from: http://apps.who.int/medicinedocs/en/d/Js21694en/. Accessed September 16, 2020

9. Cosentino F, Grant PJ, Aboyans V, Bailey CJ, Ceriello A, Delgado V, et al. 2019 ESC Guidelines on diabetes, pre- diabetes, and cardiovascular diseases developed in collaboration with the EASD. Eur Heart J. 2020;41(2):255-323. doi:10.1093/eurheartj/ehz486

10. Ministry of Health, Government of Fiji. Diabetes Management Guidelines. Third Edition.2012. Available at http://www.health.gov.fj/wp-content/uploads/2018/03/Diabetes-Management-Guidelines.pdf [Accessed 29 June 2020]

11. Ministry of Health, Ghana. Standard Treatment Guidelines, Seventh Edition, 2017. Available from: https://www.moh.gov.gh/wp- content/uploads/2020/07/GHANA-STG-2017-1.pdf: [Accessed 29 June 2020]

12. Hellenic Diabetes Association (HDA). Guidelines for the management of the diabetic patient, 2018. Athens: HDA. 2017. https://www.ede.gr/wp-content/uploads/2017/odigies.pdf. [Accessed 29 June 2020].

13. Food and Health Bureau, Hong Kong SAR Government. Hong Kong Reference Framework for Diabetes Care for Adults in Primary Care Settings. Revised edition 2018. Available from: https://www.fhb.gov.hk/pho/english/resource/files/RF_DM_full.pdf. [Accessed 29 June 2020].

14. Indian Council of Medical Research. ICMR Guidelines for Management Of Type 2 Diabetes 2018.2018

15. International Diabetes Federation Clinical Guidelines Task Force. Global Guidelines for Type 2 Diabetes, 2012. https://www.idf.org/sites/default/files/IDF%20T2DM%20Guideline.pdf [Accessed 29 June 2020]

16. International Diabetes Federation Clinical Guidelines Task Force. Global Guidelines for Type 2 Diabetes, 2017. https://www.idf.org/sites/default/files/IDF%20T2DM%20Guideline.pdf [Accessed 29 June 2020]

17. Ministry of Public Health and Sanitation, Kenya. National Clinical Guidelines for Management of Diabetes Mellitus. 2010.

18. Kim MK, Ko SH, Kim BY, Kang ES, Noh J, Kim SK, et al. 2019 Clinical Practice Guidelines for Type 2 Diabetes Mellitus in Korea. Diabetes Metab J. 2019;43(4):398-406. doi:10.4093/dmj.2019.0137

19. Guzmán JR, Lyra R, Aguilar-Salinas CA, Cavalcanti S, Escaño F, Tambasia M, Duarte E; ALAD Consensus Group. Treatment of type 2 diabetes in Latin America: a consensus statement by the medical associations of 17 Latin American countries. Latin American Diabetes Association. Rev Panam Salud Publica. 2010 Dec;28(6):463-71. doi: 10.1590/s1020-49892010001200008.

20. Latin American Diabetes Association. ALAD 2019 Guidelines on the Diagnosis, Control and Treatment of Type 2 Diabetes Mellitus with Evidence-Based Medicine (Guías ALAD 2019 para el diagnóstico y manejo de la diabetes mellitus tipo 2 con medicina basada en evidencia). J Lat Am Diabetes Assoc Extraordinary Ed 2019. http://www.revistaalad.com/guias/5600AX191_guias_alad_2019.pdf [Accessed 29 June 2020]

21. Sherif I, Kadiki O , Khdora A, Lakhdar A, Elwersheffani S, Swalem A, et al. LIBYAN DIABETES CARE GUIDELINES. 1st Edition: Libyan Ministry of Health, Libyan Board of Medical Specialization, Libyan Association for Diabetes and Endocrinology. 2010.

22. Ministry of Health Malaysia. Clinical Practice Guidelines (CPG): Management of Type 2 Diabetes Mellitus (T2DM), 2015. Ministry of Health, Putrajaya, Malaysia (2015). Available at: http://www.acadmed.org.my/ [Accessed 29 June 2020].

23. PROYECTO de Norma Oficial Mexicana PROY-NOM-015-SSA2-2018, Para la prevención, detección, diagnóstico, tratamiento y control de la Diabetes Mellitus. Available from: http://www.diariooficial.gob.mx/normasOficiales.php?codp=7112&view=si# Accessed July 16, 2020.

24. New Zealand Guidelines Group. Guidance on the Management of type 2 diabetes 2011. Wellington: New Zealand Guidelines Group; 2011. Available from: http://www.health.govt.nz/publication/new-zealand-primary-care-handbook-2012

25. Chinenye S, Onyemelukwe G, Ogbera A, Uloko, A. GUIDELINES FOR DIABETES MANAGEMENT IN NIGERIA - 2ND EDITION new corrected final one 05.2019. Available at https://www.researchgate.net/publication/330986960_GUIDELINES_FOR_DIABETES_MANAGEMENT_IN_NIGERIA_-_2ND_EDITION_new_corrected_final_one_05.

26. Ministerio de Salud del Perú. Guía de práctica clínica para el Diagnóstico, Tratamiento y Control de la Diabetes Mellitus tipo 2 en el Primer Nivel de Atención. Lima, Perú: MINSA; 2016. Available from: http://bvs.minsa.gob.pe/local/MINSA/3466.pdf

27. Unite for Diabetes Philippines. Philippine Practice Guidelines for the Diagnosis and Management of Diabetes. 2010. [cited 2017 Jun 8]. Available from: http://www.pcdef.org/philippine-clinical-practice-guidelines-for-diabetes

28. Scottish Intercollegiate Guidelines Network (SIGN). Pharmacological management of glycaemic control in people with type 2 diabetes. Edinburgh: SIGN; 2017. (SIGN publication no. 154). [November 2017]. Available from URL: http://www.sign.ac.uk

29. Ministry of Health Singapore. Clinical practice guidelines Diabetes Mellitus. MOH Clinical Practice Guidelines 1/2014. ISBN 978-981-09-0006- 9

30. The Society for Endocrinology, Metabolism and Diabetes of South Africa Type 2 Diabetes Guidelines Expert Committee (SEMDSA). The 2017 SEMDSA Guideline for the Management of Type 2 Diabetes Guideline Committee. JEMDSA 2017; 21(1) (Supplement 1): S1-S196.

31. Gomez-Peralta F, Escalada San Martín FJ, Menéndez Torre E, Mata Cases M, Ferrer García JC, Ezkurra Loiola P, et al; Spanish Diabetes Society (SED) recommendations for the pharmacologic treatment of hyperglycemia in type 2 diabetes: 2018 Update. Recomendaciones de la Sociedad Española de Diabetes (SED) para el tratamiento farmacológico de la hiperglucemia en la diabetes tipo 2: Actualización 2018. *Endocrinol Diabetes Nutr (Engl Ed)*. 2018;65(10):611-624. doi:10.1016/j.endinu.2018.08.004.

32. Somasundaram NP, Wijeyaratne CN, De Silva S, Siribaddana S, Illangasekera U, Rajaratnam H, et al. 2013. Diabetes Mellitus: Glucose Control. Sri Lanka Journal of Diabetes Endocrinology and Metabolism, 3(1), pp.45–57. DOI: http://doi.org/10.4038/sjdem.v3i1.5505.

33. Standard Treatment Guidelines and Essential Medicines List of Common Medical Conditions in the Kingdom of Swaziland. First Edition, 2012 [Internet]. Available from: http://www.gov.sz/index.php/health-documents.

34. Swedish National Board of Health and Welfare. National Guidelines for Diabetes Care. 2015. Available from: http://www.socialstyrelsen.se/nationalguidelines

35. Ministry of Health, Community Development, Gender, Elderly and Children, Tanzania. Standard Treatment Guidelines (STG) and National Essential Medicines List TANZANIA MAINLAND (NEMLIT). 2017.

36. THE SOCIETY of ENDOCRINOLOGY and METABOLISM of TURKEY (SEMT). Clinical Practice Guideline for Diagnosis, Treatment and Follow-up of Diabetes Mellitus and Its Complications - 2019. 12th Edition. 2019 Available from: http://temd.org.tr/admin/uploads/tbl_kilavuz/20191107144832-2019tbl_kilavuz7c65cb4e70.pdf

37. American Diabetes Association. Standards of Medical Care in Diabetes—2020. Diabetes Care 2020;43(Supplement 1).

38. Ministry of health, Uganda. Uganda Clinical Guidelines 2016 – National Guidelines for Management of Common Conditions. Available from: http://library.health.go.ug/publications/guidelines/uganda-clinical-guidelines-2016.

39. National Institute for Health and Care Excellence. Type 2 diabetes: The management of type 2 diabetes (NG28): National Institute for Health and Care Excellence, 2015. Available at: https://www.nice.org.uk/guidance/ng28

HIV/AIDS

40. Australasian Society for HIV. Antiretroviral guidelines: US DHHS guidelines with Australian commentary. 2019. Available at: www.arv.ashm.org.au (accessed June 2020).

41. Ministry of Health, Botswana. Handbook of the Botswana 2016 Integrated HIV Clinical Care Guidelines. 2016. Available at: https://www.moh.gov.bw/guidelines.html

42. British Columbia Centre for Excellence in HIV/AIDS (BC-CfE). (2020). THERAPEUTIC GUIDELINES ANTIRETROVIRAL (ARV) TREATMENT OF ADULT HIV INFECTION. Available at . http://www.bccfe.ca/therapeutic-guidelines

43. AIDS Hepatitis C Group, Infectious Diseases Branch, Chinese Medical Association, Chinese Center for Disease Control and Prevention. Chinese AIDS Diagnosis and Treatment Guide (2018 Edition) [J]. Chinese Journal of Internal Medicine, 2018, 57 (12): 867-884. DOI: 10.3760 / cma.j.issn.0578-1426.2018.12.002

44. Federal Ministry of Health, Ethiopia. NATIONAL CONSOLIDATED GUIDELINES FOR COMPREHENSIVE HIV PREVENTION, CARE AND TREATMENT. 2018. Available at: https://www.afro.who.int/sites/default/files/2019-04/National%20Comprehensive%20HIV%20Care%20%20Guideline%202018.pdf.

45. European AIDS Clinical Society Guidelines version 10.1. 2020. Available at: https://www.eacsociety.org/guidelines/eacs-guidelines/eacs-guidelines.html. (Accessed November 2020).

46. National AIDS/STI Control Programme (NACP), Ghana Health Service. GUIDELINES FOR ANTIRETROVIRAL THERAPY IN GHANA. 2016. Sixth Edition. Accra, Ghana: GOG. Available at: http://www.ccmghana.net/index.php/policies- guidelines?download=199:art-guidelines-revised-2017. (Accessed November 2020).

47. National AIDS Control Organization, Ministry of Health and Family Welfare, Government of India. NATIONAL TECHNICAL GUIDELINES ON ANTI RETROVIRAL TREATMENT. 2018. Available at: http://naco.gov.in/documents/policy-guidelines

48. World Health Organization. (2016). Consolidated guidelines on the use of antiretroviral drugs for treating and preventing HIV infection: recommendations for a public health approach, 2nd ed. World Health Organization. https://apps.who.int/iris/handle/10665/208825

49. Ministry of Health, Jamaica. CLINICAL MANAGEMENT OF HIV DISEASE: Guidelines for Medical Practitioners. 2017. Available at: https://moh.gov.jm/wp-content/uploads/2015/03/Clinical-Management-of-HIV-Disease-2017.pdf

50. Ministry of Health, National AIDS & STI Control Program. Guidelines on Use of Antiretroviral Drugs for Treating and Preventing HIV Infection in Kenya 2018 Edition. Nairobi, Kenya: NASCOP, August 2018. Print.

51. Korean Society for AIDS. The 2018 Clinical Guidelines for the Diagnosis and Treatment of HIV/AIDS in HIV-Infected Koreans. Infect Chemother. 2019;51(1):77-88. doi:10.3947/ic.2019.51.1.77

52. Ministry of Health, Republic of Liberia. National Standard Therapeutic Guidelines and Essential Medicines List, 2nd Edition, 2017. Available from: https://moh.gov.lr/wp-content/uploads/Liberia-NSTG-EML-2nd-Edition-2017.pdf: [Accessed 29 June 2020].

53. Ministry of Health, Malawi. Guidelines for Clinical Management of HIV in Children and Adults, 3rd Edition, 2016. Available from: https://www.childrenandaids.org/sites/default/files/2017-04/Malawi_Clinical-HIV-Guidelines_2016.pdf: [Accessed 29 June 2020]

54. Koh, Kwee. (2017). Malaysian Consensus Guidelines on Antiretroviral Therapy 2017 MINISTRY OF HEALTH MALAYSIA.

55. Federal Ministry of Health (FMOH). Standard Treatment Guideline. 2nd ed. Abuja, Nigeria: Federal Ministry of Health; 2016. Available from: http://www.health.gov.ng/doc/FMOH%20-%20Nigeria%20Standard%20Treatment%20Guidelines%202nd%20Edition%20(2016).pdf: [Accessed 29 June 2020].

56. National HIV Programme for Ministry of Health/National Center for Infectious Diseases, Singapore. Recommendations for the Use of Antiretroviral Therapy in Adults Living with HIV in Singapore; 2019. Available at https://www.ncid.sg/About-NCID/OurDepartments/Documents/ART%20Recommendations.pdf

57. National Department of Health, Republic of South Africa. 2019 ART Clinical Guidelines for the Management of HIV in Adults, Pregnancy, Adolescents, Children, Infants and Neonates. Pretoria, South Africa: Department of Health, Republic of South Africa, 2019. Available at:

https://sahivsoc.org/SubHeader?slug=ndoh-and-who- guidelines.

58. Ministry of Health, Sri Lanka. The Guideline Use of Antiretroviral Drugs for Treating and Prevention of HIV Infection. 2014. Available at: http://www.aidscontrol.gov.lk/images/pdfs/publications/guidelines/sri_lankan_art_guidelines-for_prev_and-treatment_of_hiv_2014_web.pdf.

59. Ministry of Health, Kingdom of Swaziland. Integrated HIV Management Guidelines. 2015. Available at: http://swaziaidsprogram.org/standards-guidelines/.

60. Eriksen J, Albert J, Blaxhult A, Carlander C, Flamholc L, et al. Antiretroviral treatment for HIV infection: Swedish recommendations 2016. Infect Dis (Lond). 2017 Jan;49(1):1-34. doi: 10.1080/23744235.2016.1247495.

61. Panel on Antiretroviral Guidelines for Adults and Adolescents. Guidelines for the Use of Antiretroviral Agents in Adults and Adolescents with HIV. Department of Health and Human Services. Available at http://www.aidsinfo.nih.gov/ContentFiles/ AdultandAdolescentGL.pdf. Accessed [29 June 2020]

62. British HIV Association, BHIVA guidelines for the routine investigation and monitoring of adult HIV-1-positive individuals 2016 (2019 interim update) Available at: (https://www.bhiva.org/monitoring-guidelines)

63. Ministry of Health, Republic of Zambia. Zambia Consolidated Guidelines for Treatment and Prevention of HIV Infection. 2020. Available at: https://www.nac.org.zm/sites/default/files/publications/Consolidated%20Guidelines%202020.pdf.

64. The National Medicine and Therapeutics Policy Advisory Committee (NMTPAC), Ministry of Health and Child Care (MOHCC), Zimbabwe. Guidelines for Antiretroviral Therapy for the Prevention and Treatment of HIV in Zimbabwe. 2016. Available at: https://depts.washington.edu/edgh/zw/vl/project-resources/ZIM_ART_Guidelines_2016_-_review_final.pdf.

Hypertension

65. Ministry of Public Health, Islamic Republic of Afghanistan. National Standard Treatment Guidelines for the Primary Level. 2013. Available at: https://moph.gov.af/sites/default/files/2019-07/NSTG%20English.pdf

66. Delucchi, A, Marín, M, Páez, O, Bendersky, M, Rodríguez, P, & en representación de los participantes designados por la Sociedad Argentina de Cardiología, Federación Argentina de Cardiología y Sociedad Argentina de Hipertensión Arterial (2019). Principales conclusiones del Consenso Argentino de Hipertensión Arterial [Principal conclusions of the Argentine Consensus on Arterial Hypertension]. Hipertension y riesgo vascular, 36(2), 96–109. https://doi.org/10.1016/j.hipert.2019.01.001

67. National Heart Foundation of Australia. Guideline for the diagnosis and management of hypertension in adults – 2016. Melbourne: National Heart Foundation of Australia, 2016.

68. Al-Saweer A. Hypertension in clinical practice. Educatin-family physician corner.Bahrain Medical Bulletin.2011;33(1):38-43

69. Hypertension Task Group (2011) Hypertension Guidelines for Bermuda Government of Bermuda: Department of Health

70. Ministry of Health, Republic of Botswana. Practical Approach to Care Kit (PACK) - Botswana Primary Care Guideline for Adult. 2013. Available at: https://knowledgetranslation.co.za/wp-content/uploads/2018/04/PACK-Botswana-

Adult-2013-SAMPLE.pdf

71. Malachias MVB, Andrea Araujo Brandão AA, Kaiser S, Moreira O Filho. "th Brazilian Guideline of Arterial Hypertension: Chapter 5 - Therapeutic Decision and Targets. Arq Bras Cardiol. 2016 Sep;107(3 Suppl 3):25-29. doi: 10.5935/abc.20160155.

72. Nerenberg KA, Zarnke KB, Leung AA, Dasgupta K, Butalia S, et al; Hypertension Canada's 2018 Guidelines for Diagnosis, Risk Assessment, Prevention, and Treatment of Hypertension in Adults and Children. Can J Cardiol. 2018 May;34(5):506-525. doi: 10.1016/j.cjca.2018.02.022. Epub 2018 Mar 1.

73. Joint Committee for Guideline Revision. 2018 Chinese Guidelines for Prevention and Treatment of Hypertension-A report of the Revision Committee of Chinese Guidelines for Prevention and Treatment of Hypertension. J Geriatr Cardiol. 2019;16(3):182-241. doi:10.11909/j.issn.1671-5411.2019.03.014

74. Cosentino F, Grant PJ, Aboyans V, Bailey CJ, Ceriello A, Delgado V, et al. 2019 ESC Guidelines on diabetes, pre-diabetes, and cardiovascular diseases developed in collaboration with the EASD. Eur Heart J. 2020 Jan 7;41(2):255-323. doi: 0.1093/eurheartj/ehz486.

75. Ministry of Health, Government of Fiji. Cardiovascular Therapeutic Guidelines. Third Edition. 2015. Available at http://www.health.gov.fj/wp-content/uploads/2018/03/Diabetes-Management-Guidelines.pdf [Accessed 29 June 2020]

76. Food and Health Bureau, Hong Kong SAR Government. Hong Kong Reference Framework for Hypertension Care for Adults in Primary Care Settings. Revised edition 2018. Available from: https://www.fhb.gov.hk/pho/english/resource/files/RF_HT_full.pdf. Accessed 15 May 2020.

77. Shah SN, Munjal YP, Kamath SA, Wander GS, Mehta N, Mukherjee S, et al. Indian guidelines on hypertension-IV (2019). J Hum Hypertens. 2020;34(11):745-758. doi:10.1038/s41371-020-0349-x

78. Unger T, Borghi C, Charchar F, Khan NA, Poulter NR, Prabhakaran D, et al. 2020 International Society of Hypertension Global Hypertension Practice Guidelines. Hypertension. 2020;75(6):1334-1357. doi:10.1161/HYPERTENSIONAHA.120.15026

79. Ministry of Health, Jamaica. GUIDELINES FOR THE MANAGEMENT OF HYPERTENSION. 2014. Available at: https://extranet.who.int/ncdccs/Data/JAM_D1_Hypertension%20Guidelines%20Revised%20Version%20March%202014%20final%20version%202.pdf.

80. Division of Non-Communicable Diseases - Ministry of Health, Kenya. KENYA NATIONAL GUIDELINES FOR CARDIOVASCULAR DISEASES MANAGEMENT. 2018.

81. Lee H-Y, Shin J, Kim G-H, et al. 2018 Korean Society of Hypertension Guidelines for the management of hypertension: part II-diagnosis and treatment of hypertension. Clin Hypertens. 2019;25. doi:10.1186/s40885-019-0124-x

82. Task Force of the Latin American Society of Hypertension Guidelines on the management of arterial hypertension and related comorbidities in Latin America, Journal of Hypertension: August 2017 - Volume 35 - Issue 8 - p 1529-1545 doi: 10.1097/HJH.0000000000001418

83. Ministry of Health, Malawi. Malawi Standard Treatment Guidelines (MSTG). 5th Edition. 2015. Available at: http://www.medcol.mw/wp- content/uploads/2015/12/Malawi-Standard-Treatment-Guidelines-Essential- Medicines-List-2015.pdf

84. Ministry of Health Malaysia, Academy of Medicine of Malaysia & Malaysian Society of Hypertension. Clinical practice guidelines: management of hypertension 5th ed. Available

at: http://www.moh.gov.my. Accessed 30 Jul 2020.

85. Scottish Intercollegiate Guidelines Network (SIGN). SIGN 149 - Risk estimation and the prevention of cardiovascular disease. A national clinical guideline. 2017; Available from: https://www.sign.ac.uk/assets/sign149.pdf

86. Ministry of Health Singapore. Clinical practice guidelines Hypertension. MOH Clinical Practice Guidelines 1/2017. ISBN 978-981-11-5731-8

87. Hypertension guideline working group, Seedat YK, Rayner BL, Veriava Y. South African hypertension practice guideline 2014. Cardiovasc J Afr. 2014 Nov-Dec;25(6):288-94. doi: 10.5830/CVJA-2014-062.

88. Ceylon College of Physicians, Sri Lanka. CLINICAL PRACTICE GUIDELINES: HYPERTENSION MANAGEMENT GUIDELINES. 2016. Available at: https://www.ccp.lk/guidlines

89. Whelton PK, Carey RM, Aronow WS, Casey DE Jr, Collins KJ, Dennison Himmelfarb C, et al. 2017 ACC/AHA/AAPA/ ABC/ACPM/AGS/APhA/ASH/ASPC/NMA/PCNA guideline for the prevention, detection, evaluation, and management of high blood pressure in adults: a report of the American College of Cardiology/ American Heart Association Task Force on Clinical Practice Guidelines. Hypertension. 2018;71:e13–115

90. National Institute for Health and Care Excellence. Hypertension in adults: diagnosis and management (NG136). National Institute for Health and Care Excellence, 2019. Available at: https://www.nice.org.uk/guidance/ng136
